# Bioaccessibility and Microbial Metabolism of (Poly)phenol-Rich Dietary Fiber Fractions from Frozen Mixed Berries in an *In Vitro* Model

**DOI:** 10.1021/acs.jafc.6c01779

**Published:** 2026-10-21

**Authors:** Vanesa Núñez-Gómez, José Luis Ordóñez-Díaz, Jose Manuel Moreno-Rojas, Gema Pereira-Caro, María Jesús Periago, Rocío González-Barrio

**Affiliations:** † Department of Food Technology, Food Science and Nutrition, 16751 University of Murcia, Campus Mare Nostrum, Campus de Espinardo, 30100 Murcia, Spain; ‡ Biomedical Research Institute of Murcia Pascual Parrilla−IMIB, 30120 Murcia, Spain; § Area of Agroindustry and Food Quality, Andalusian Institute of Agricultural and Fisheries Research and Training (IFAPA), Alameda del Obispo, Avda. Menéndez-Pidal, 14004 Córdoba, Spain; ∥ Foods for Health Group, Instituto Maimónides de Investigación Biomédica de Córdoba (IMIBIC), 14004 Córdoba, Spain

**Keywords:** gastrointestinal digestion, fecal fermentation, dietary fiber, HPLC-HRMS analysis, SCFAs, antioxidant capacity, prebiotic effect

## Abstract

Berries are rich in (poly)­phenols and dietary fiber (DF), both known for their health-promoting properties. However, little is known about how these compounds interact within the berry matrix. This study characterized berry fiber fractions as value-added ingredients and assessed their effects on (poly)­phenol bioaccessibility and fecal microbiota metabolism. Three fractions were obtained from freeze-dried berries (FDB): insoluble fiber (IFF), soluble fiber (SFF), and (poly)­phenol-rich extract (PRE). IFF had the highest DF content and was rich in anthocyanins and ellagic acid. *In vitro* fecal fermentation showed that all fractions promoted short-chain fatty acid production: SFF generated the most acetate, IFF the most propionate, and FDB the most butyrate. Microbiota-derived metabolites from flavan-3-ols and ellagic acid peaked in IFF at 8–24 h, suggesting gradual release from the fiber matrix. These findings highlight the novelty of using fiber–(poly)­phenol complexes as potential prebiotic carriers for controlled delivery of bioactives in the colon.

## Graphical abstract

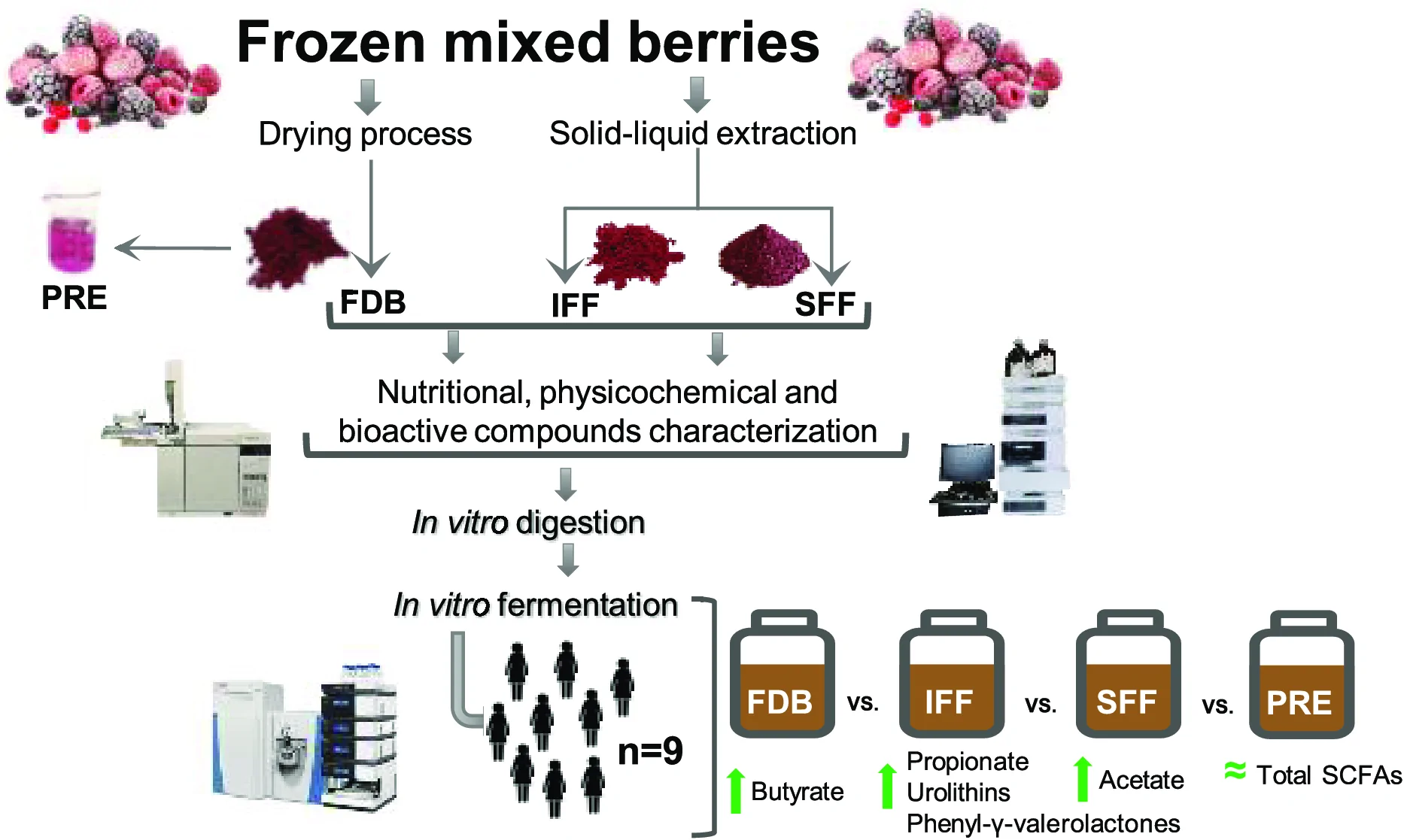

## Introduction

Berries are particularly important for their nutritional and health-promoting properties and are widely consumed globally. Berries such as strawberries, blackberries, raspberries, and blueberries are rich in dietary fiber (DF), vitamins (A, C, E, and B), and minerals (calcium, sodium, phosphorus, iron, and potassium).^1^ They also contain non-nutritive compounds with important health-promoting properties, such as (poly)­phenols, including tannins, phenolic acids, flavonoids, and stilbenes, among others.^1^ Numerous studies have attributed a range of health benefits to these compounds, including cardiovascular protection, antimicrobial, antioxidant, anti-inflammatory, antitumor, antiaging, and neuroprotective effects.^2^

Based on their composition, berries may exert potential prebiotic effects; however, these depend on the specific characteristics of their DF and (poly)­phenol composition.^3^ The DF in berries, reaches the colon where it undergoes fermentation by the fecal microbiota. Consequently, short-chain fatty acids (SCFAs) such as acetate, propionate, and butyrate are produced as end products of bacterial metabolism. These metabolites can serve as energy sources for bacteria, inhibit the growth of pathogenic organisms, modulate intestinal immunity, and diminish inflammatory responses.^4^

Several studies have highlighted that dietary (poly)­phenols exhibit a “duplibiotic effect”, a dual mechanism that promotes gut health.^5,6^ They selectively inhibit potential pathogens, while acting as prebiotic-like substrates that stimulate the growth of beneficial gut bacteria. Moreover, the positive impact of (poly)­phenols on host health is attributed to their ability to reduce proinflammatory responses and mitigate metabolic syndrome, among other mechanisms. The production of gut microbiota-derived metabolites from (poly)­phenols also contributes to this effect.^5,6^ However, the microbiota’s metabolic activity toward individual (poly)­phenols is quite specific. Of particular importance are urolithins and valerolactones, which are derived from ellagic acid and ellagitannins, and flavan-3-ols, respectively, through microbiota metabolism. These gut microbiota-derived metabolites are known to enhance cellular and gut health, increase mitochondrial function and mitophagy, reduce inflammation, and protect against age-related diseases.^7,8^

While the potential prebiotic properties of DF and (poly)­phenols from berries have been extensively documented, these effects depend on the specific compounds present, the microbial functions involved, and the composition of the fecal microbiota.^3^ In this context, a significant knowledge gap remains regarding the functional behavior of berry-derived fractions, in which these components are naturally embedded within a complex food matrix. Since the physical and chemical interactions between DF and (poly)­phenols may modify the physicochemical characteristics of the matrix, modulate the release and bioaccessibility of (poly)­phenols, and influence microbial fermentation dynamics. These interactions may affect both the production of SCFAs and the formation of phenolic catabolites. Nonetheless, the gastrointestinal fate and fermentative behavior of such polyphenol-rich DF fractions remain incompletely characterized, limiting the comprehensive assessment of their prebiotic potential and health implications.

Therefore, this study aimed to comprehensively characterize (poly)­phenol-rich DF fractions isolated from mixed frozen berries, focusing on their chemical composition, hydration properties, and interactions between DF and (poly)­phenols. Furthermore, we investigated the metabolic fate of these complex matrices during *in vitro* gastrointestinal digestion and fecal fermentation assessing the production of SCFAs and the formation of microbial-derived phenolic metabolites.

## Materials and Methods

### Extraction Process of Dietary Fiber-Rich Fractions and Extract

Two fractions with high DF content were obtained from a mixture of frozen berries (25% strawberries, 20% blackberries, 20% blackcurrants, 20% redcurrants, 10% raspberries, and 5% blueberries) (Figure S1). The procedure used for the extraction was based on a previously published method.^9^ Prior to defrosting, the berries were transferred to a chamber at −80 °C and freeze-dried (0.05 mbar, −50 °C) using a Lyoquest freeze-dryer (Telstar, Bensalem, PA, USA) to obtain freeze-dried berries (FDB). The mixture was then homogenized by grinding in a Thermomix TM-31 (Vorwerk, Wuppertal, Germany). The resulting mixture was dissolved in water at a water-to-sample ratio of 2.5:1 (v/m) and stirred for 30 min. It was then centrifuged at 4500*g* for 5 min at room temperature in a Beckman J2–21 centrifuge (Indianapolis, USA). The resulting pellet was placed on a stove at 45 °C until it reached a constant weight, which corresponded to the insoluble fiber-rich fraction (IFF). The supernatant from the last extraction, which contained water-soluble compounds, was collected, mixed with absolute ethanol (5:1, v/v), and left overnight at room temperature to precipitate the soluble fiber. The mixture was then separated by decantation, and the solid phase was freeze-dried, which corresponded to the soluble fiber-rich fraction (SFF).

An extract rich in (poly)­phenols (PRE) was obtained from FDB by adding 0.5 g of the freeze-dried material to a solution of methanol, water, and formic acid (79/20/1, v/v/v). After 1 h of rotational agitation in the dark, the solution was evaporated and resuspended in water. This extract was prepared for *in vitro* fecal fermentation to study the prebiotic-like effect of (poly)­phenols analyzing the production of SCFAs.

### Dietary Fiber Content, Composition and Hydration Properties

Total fiber (TF), insoluble fiber (IF), and soluble fiber (SF) were determined in FDB, IFF and SFF. PRE was not analyzed because the extraction process is expected to remove most insoluble and soluble fiber fractions. Therefore, fiber characterization was focused on the fractions containing the main structural carbohydrate components (FDB, IFF and SFF). However, the absence of PRE characterization does not allow the complete exclusion of residual oligosaccharides or other fermentable carbohydrates as potential contributors to the effects studied. The enzymatic and gravimetric method described by Prosky et al.^10^ was performed using a Fibertec E 1023 system (Höganäs, Sweden). The composition of DF in the fractions was analyzed by measuring the percentage of neutral sugars in TF via GLC-FID using the Englyst method.^11^ The percentage of uronic acids was also determined using a colorimetric method described by Scott,^12^ as detailed in a previous work.^9^ These results enabled us to estimate the percentages of pectin, hemicellulose, and cellulose in the fractions, using the calculations proposed by Houben et al. and by Umaña., Eim-Iznardo, and Roselló.^13,14^ The water retention (WRC) and swelling capacities (SWC) of the fractions were evaluated using the method previously described by Navarro-González et al.^15^

### Antioxidant Capacity and (Poly)­phenol Composition

The antioxidant capacity was assessed using the ferric reducing antioxidant power (FRAP) and oxygen radical antioxidant capacity (ORAC) methods, following the procedures outlined by Benzie & Strain^16^ and Ou et al.,^17^ respectively. Results were expressed as μmol of Trolox equivalents (TE)/g. The antioxidant capacity methods were adapted for a microplate spectrophotometer (BioTek Instruments, Winooski, VT, USA), and the total content was expressed as the sum of the EPP and NEPP fractions.

Extraction of both the extractable (EPP) and nonextractable (NEPP) (poly)­phenols was conducted separately using a modified version of the method described by Arranz et al.,^18^ as detailed in our previous work.^9^ For the EPP, MeOH/H2O/formic acid (79/20/1, v/v/v) was added to each sample, followed by shaking and centrifugation. The supernatant was dried under vacuum, redissolved in acidified Milli-Q water, loaded onto a preconditioned Sep-Pak C18 cartridge, and the compounds were recovered in MeOH. For the NEPP extraction, the remaining pellet was mixed with MeOH/H2SO4 (9/1, v/v) and incubated at 85 °C for 20 h in a shaking incubator. The resulting supernatant was then processed as described above for the EPP and recovered in MeOH.

The HPLC method described by González-Barrio et al.^19^ was used to identify individual (poly)­phenols in both EPP and NEPP fractions of the samples. Identification was performed on an Agilent 1200 instrument (Waldbronn, Germany) with a scanning range of 200 to 600 nm. For the analysis, a LiChroCART RP-18 column (250 × 4.6 mm, i.d. 5 μm) was used in conjunction with a precolumn (4 × 4 mm) made of the same material (Merck, Darmstadt, Germany). The mobile phases, 1% formic acid (A) and acetonitrile (B), were delivered at a flow rate of 1 mL/min. Anthocyanins were quantified by comparison with the standard cyanidin-3-*O*-glucoside at 520 nm; hydroxycinnamic acid derivatives at 320 nm as chlorogenic acid; ellagic acid derivatives at 360 nm as ellagic acid; and ellagitannins and flavan-3-ols at 280 nm, using punicalagin and catechin as the standards, respectively. The results were expressed in mg/100 g dry weight (d.w.).

### *In Vitro* Digestion and Fecal Fermentation

To investigate the potential impact of DF on the metabolism of (poly)­phenols in the different fractions (FDB, IFF, SFF, and PRE), the samples underwent *in vitro* digestion using the INFOGEST protocol.^20^ After digestion, the samples were lyophilized and subsequently subjected to *in vitro* fecal fermentation (for 48 h to capture full (poly)­phenol metabolism given colonic transit time variability^21^) using a pooled fecal inoculum from nine healthy, normal-weight women volunteers aged 25–35 years. The volunteers were required to follow specific nutritional restrictions for 2 days prior to the study, which excluded all sources of fiber and (poly)­phenols from the diet, as indicated in our previous work.^9^ All participants provided written informed consent prior to their inclusion in the study. The study received ethical approval from the Research Ethics Commission (2664/2019) of the University of Murcia (Spain). The fermentation process was based on the method described by Jaganath et al.^22^ and adapted from González-Barrio et al.^23^ (Extended version in Supporting Information).

To assess the potential prebiotic activity of the samples, the production of short-chain fatty acids (SCFAs) - namely acetate, propionate, and butyrate - was measured. The SCFAs were analyzed in fecal slurries collected at 0, 4, 8, 24, and 48 h by GC-FID, following the protocol described by Baenas et al.^24^ The SCFAs were identified by comparing their retention times with those of authentic standards and quantified using calibration curves constructed with a Volatile Free Acid mix (Supelco, Pennsylvania, USA). The concentrations were expressed in millimolar (mM) units.

### Analysis by UHPLC-HRMS of Fecal Microbiota-Derived Metabolites of (Poly)­phenol

The fermented samples were subjected to (poly)­phenol extraction and analysis as described by Núñez-Gómez et al.^25^ In short, the extracted (poly)­phenols were analyzed on an Ultimate 3000 RS UHPLC system with a Zorbax SB-C18 RRHD column (100 × 2.1 mm i.d., 1.8 μm) maintained at 40 °C, following the protocol of Ordoñez-Díaz et al.^26^ Components were separated using a gradient of 0.1% water with formic acid (A) and acetonitrile/formic acid (99.9/0.1, v/v) (B) as mobile phases. Analytes were detected by an Exactive Orbitrap mass spectrometer (ThermoFisher Scientific, San José, CA, USA) operating in negative ionization mode. Data acquisition and processing were performed using Xcalibur 3.0 software. Identified substances were classified according to the Metabolite Standards Initiative Metabolite Identification Level (MSIMI) and quantified using calibration curves.^27^ Quantification of phenolic metabolites was performed using the exact mass of the molecular ion and external calibration curves. In the absence of reference standards, quantification was carried out using calibration curves of structurally related compounds. The limits of detection and quantification ranged from 0.03 to 1.03 μg/mL and from 0.09 to 3.1 μg/mL, respectively. When authentic standards were unavailable, compounds were tentatively identified by comparing their exact mass and retention time with those of compounds in databases such as Metlin (https://metlin.scripps.edu) and Phytohub (https://phytohub.eu/).

### Statistical Analysis

Statistical analyses were conducted in RStudio version 4.0.5. Data normality and homogeneity of variances were assessed using the Shapiro–Wilk and Bartlett tests, respectively. One-way ANOVA was used to assess significant differences among samples, and two-way ANOVA was used to evaluate metabolite production during fecal fermentation, considering sample type and fermentation time. Tukey’s test was used for post hoc comparisons, with significance set at *p*-value < 0.05. When normality assumptions were not met, the Kruskal–Wallis test was used. Additionally, correlation analyses were conducted among physicochemical properties, fiber composition parameters, and SCFAs.

## Results and Discussion

### Dietary Fiber Characterization and Hydration Properties

Following the procedure illustrated in Figure S1, we obtained two fiber fractions (IFF and SFF) and freeze-dried berries (FDB). The samples were chemically characterized, and the results are shown in Table 1.

**Table 1:** Dietary Fiber Content and Proportion of Neutral Sugars, Uronic Acid, Cellulose, Hemicellulose and Pectin in Berry Samples Expressed in Percentage (%) and Hydration Properties[Table-fn t1fn1]

	FDB	IFF	SFF
total dietary fiber (TF)	27.4 ± 0.2^c^	65.1 ± 1.4^a^	47.4 ± 2.3^b^
insoluble dietary fiber (IF)	22.9 ± 0.5^b^ (84%)[Table-fn t1fn2]	56.4 ± 0.5^a^ (87%)	2.6 ± 0.6^c^ (5%)
soluble dietary fiber (SF)	4.5 ± 0.7^b^ (16%)	8.7 ± 0.9^b^ (13%)	44.8 ± 2.9^a^ (95%)
rhamnose	0.9 ± 0.1^c^	1.2 ± 0.2^b^	2.3 ± 0.1^a^
arabinose	7.3 ± 0.5^b^	8.7 ± 0.6^a^	5.8 ± 0.2^c^
xylose	17.4 ± 0.7^b^	22.1 ± 2.2^a^	nd
mannose	13.0 ± 2.6^b^	18.5 ± 1.9^a^	1.3 ± 0.2^c^
galactose	7.3 ± 2.7	7.1 ± 0.3	5.3 ± 0.2
glucose	16.1 ± 0.7^a^	7.9 ± 1.9^b^	6.0 ± 0.6^b^
uronic acid	38.1 ± 2.4^b^	34.5 ± 3.5^b^	79.4 ± 0.4^a^
cellulose[Table-fn t1fn3]	14.5 ± 0.6^a^	7.1 ± 1.7	-
hemicellulose[Table-fn t1fn4]	32.0 ± 3.1^b^	41.5 ± 1.5^a^	7.4 ± 0.5^c^
pectin[Table-fn t1fn5]	53.6 ± 3.6^b^	51.5 ± 3.1^b^	92.7 ± 0.5^a^
WRC (g of water/g)	1.70 ± 0.0^b^	1.5 ± 0.1^b^	4.8 ± 0.0^a^
SWC (mL of water/g)	8.9 ± 0.9^b^	7.2 ± 0.5^c^	12.9 ± 1.0^a^

* Values are expressed as mean ± SD (*n* = 4).

# In parentheses the % with respect to total dietary fiber. Different letters (a–c) indicate significant differences (*p* < 0.05) among the samples. Freeze-dried berries (FDB); insoluble fiber fraction (IFF); soluble fiber fraction (SFF); nd (not detected); - (not calculated); water retention capacity (WRC); swelling capacity (SWC).

A Cellulose: Glucose × 0.9.

B Hemicellulose: (Fucose + Xylose + Mannose + (Glucose × 0.1)).

C Pectin: (Rhamnose + Arabinose + Galactose + Uronic acids).

Regarding fiber composition, the fiber content in FDB was consistent with data from the United States Department of Agriculture (USDA) database^28^ for individual berries included in the mixture, ranging from 20% to 66%. Notably, FDB contained 84% insoluble fiber (IF) and 16% soluble fiber (SF) (Table 1). Similar proportions have been reported by other researchers, with 22% SF in blackberries^29^ and 30% SF in raspberries,^24^ consistent with our previous findings. Isolating the IFF and SFF fractions resulted in total fiber (TF) concentrations 1.7 and 2.4 times higher than in FDB, respectively. This result is expected, as the fiber isolation protocol concentrates fiber components while removing soluble nonfiber material from the starting berry mixture. The extraction method produced distinct compositions for the IFF and SFF fractions, with IFF containing a high proportion of IF (87%) and SFF containing a high proportion of SF (95%). The IF/SF ratio observed in IFF (6.5) aligns with ratios reported by other researchers for various berry byproducts, including 7.5, 4.4, and 10 for bilberry press cake, blueberry puree, and cranberry fiber, respectively.^30^

Regarding fiber composition, the SFF fractions showed the greatest differences, primarily due to their higher uronic acid content, indicating that more pectin was isolated during extraction. These findings are consistent with those reported by Ahmadi et al.,^31^ who reported uronic acid contents of 47 and 65% for pectin extracted from Goji berry and raspberry, respectively. Results from a previous study showed that the SF fraction from raspberry, although present in higher amounts, exhibited the same profile as SFF, with uronic acids, glucose, arabinose, and galactose as the main components.^24^

Cellulose and hemicellulose are two primary components of IF.^32^ The FDB fraction exhibited the highest cellulose content, which was primarily glucose, whereas in the SFF fraction, glucose was primarily present as part of soluble polysaccharides such as pectin and, to a lesser extent, as hemicellulose. Conversely, the SFF fraction displayed the highest pectin content, 1.7 times greater than that of the other two fractions, which showed no significant differences between them. These findings were attributed to the extraction method employed, in which soluble polysaccharides were precipitated with ethanol, enabling the isolation of the pectin content.

Both WRC and SWC were related to hydration characteristics and the ability of fractions to retain water within their fibrous structure. The SFF fractions displayed the highest WRC and SWC, surpassing FDB and IFF by 1.5- and 1.8-fold, respectively. The WRC data closely mirrored values reported for pectin extracted from strawberries, blackberries, and raspberries, with mean values of 2.5, 4.2, and 2.4 g of water/g.^33^ Similarly, different berry pomace powders exhibited SWC values ranging from 5.5 to 7.1 mL of water/g.^34^ These findings suggested that the SFF fraction had the potential to significantly increase stool volume and reduce fecal transit time in the large intestine, thereby lowering the risk of intestinal diseases.^35^

### (Poly)­phenol Profile and Antioxidant Capacity of the Berry Fractions

It is widely recognized that berry pomaces are rich in (poly)­phenols, although the overall quantity and profile can vary by berry type. In the present study, the samples were obtained from a mixture of different berries. Therefore, comparisons with studies using individual berry sources should be interpreted with caution. Various studies have reported the following average values: 0.25 g/100 g of dry weight in Saskatoon berry,^36^ 10.5 g/100 g of dry weight for chokeberry, 1 g/100 g of dry weight for black chokeberry, and 4 g/100 g of dry weight for strawberries.^37^ Importantly, when fiber-rich fractions derived from pomace were extracted, certain (poly)­phenols remained in the extracted fraction due to their binding to fiber components. Consequently, both EPP and NEPP were examined in the berry fractions, including FDB, IFF, and SFF.

In all samples, the primary compounds in EPP were flavan-3-ols and anthocyanins (Figure 1a), with concentrations exceeding those reported for saskatoon berry pomace by De Souza et al.,^36^ who reported mean values of 58 mg of flavanols/100 g of dry pomace and by Tumbas Šaponjac et al.,^38^ who reported mean values ranging from 52 to 182 mg of anthocyanins/100 g of dry weight. The second most prevalent compounds were ellagic acid and ellagitannins. Among the analyzed fractions, IFF had the highest extractable ellagic acid content, whereas SFF had the lowest, a difference attributable to its extraction process (Figure 1a).

**Figure 1:**
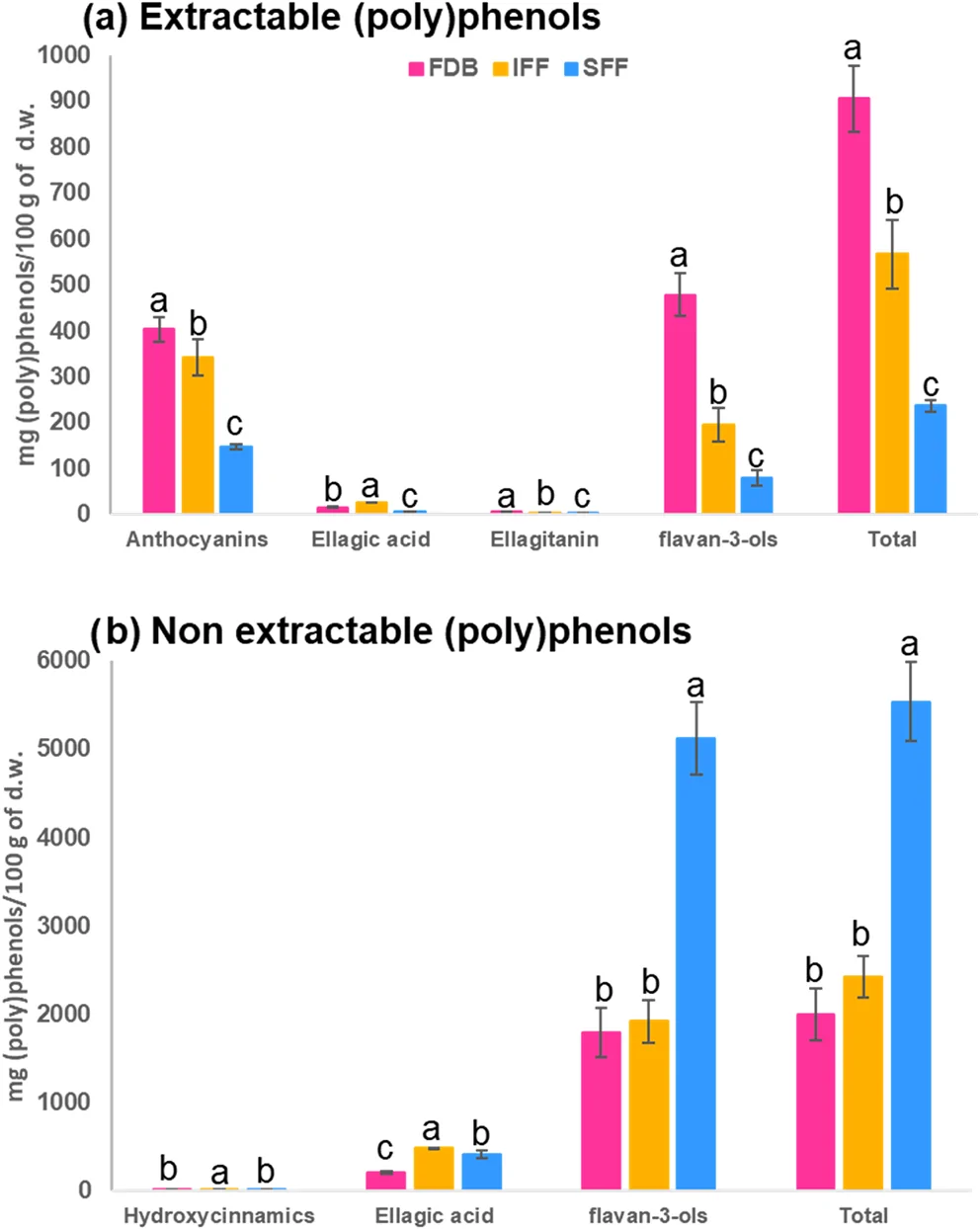
Contents of individual (poly)­phenols (anthocyanins, ellagic acid, ellagitannins, flavan-3-ols and hydroxycinnamic acid) and the total (poly)­phenols content, determined as the sum of the individual families (mg/100 g of d.w.), for (a) extractable (poly)­phenols and (b) nonextractable (poly)­phenols, analyzed by HPLC-DAD in freeze-dried berries (Pink box solid FDB), insoluble fiber-rich fraction (Yellow box solid IFF) and soluble fiber-rich fraction (Blue box solid SFF). Values are expressed as mean ± SD (*n* = 3). Different letters (a–c) indicate significant differences (*p* < 0.05) among the samples for individual and total (poly)­phenols.

In NEPP, flavan-3-ols, ellagic acid and hydroxycinnamic acids were identified, being flavan-3-ols the predominant compounds in all samples (see Figure 1b). Moreover, SFF exhibited the highest levels of flavan-3-ols and IFF the highest levels for the other two compounds.^39^ This variation is due not only to the fact that these two fiber-rich fractions have (poly)­phenols bound to the fiber molecules, but also to a mass-balance effect: when expressed per gram of sample, freeze-dried berries contain additional components such as sugars and other water-soluble compounds that dilute the fiber-associated (poly)­phenols, whereas the IFF and SFF fractions are more concentrated in dietary fiber. As a result, these fractions have a particularly interesting composition and potential health-related properties, as documented by previous studies.^40^

Tomas et al.^41^ demonstrated that adding pectin during *in vitro* digestion and fermentation of blackberry puree significantly increased NEPP and consequently reduced EPP. These compounds formed complexes with fiber molecules, producing an effect similar to that observed in our study. Notably, the total (poly)­phenol content, calculated as the sum of EPP and NEPP, was similar in IFF and FDB, whereas SFF exhibited approximately two times higher levels than the other two samples. These findings are consistent with those reported by Reynoso-Camacho et al.^42^ for a byproduct of the strawberry and blueberry decoction process, which exhibited a higher NEPP content. Conversely, byproducts of raspberries and blackberries predominantly contained EPP. This variation may be attributed to the fiber content in these byproducts, which was not specified in the publication.

Regarding antioxidant capacity (Table S1), IFF showed higher values (53.6 ± 7.7 μmol TE/g d.w. (FRAP) and 249.8 ± 6.2 μmol TE/g d.w. (ORAC)) than SFF (27.6 ± 7.8 μmol TE/g d.w. and 161.1 ± 11.0 μmol TE/g d.w., respectively) and FDB (35.8 ± 0.8 μmol TE/g d.w. and 235.6 ± 3.7 μmol TE/g d.w., respectively), consistent with findings reported by Xu et al.^43^ for IF of lychee pulp, which are attributed to a high concentration of bound (poly)­phenols. Furthermore, the FRAP activity of IFF was comparable to that reported by Azman et al.^44^ for blackcurrant pomace (58 μmol TE/g). These results are consistent with those reported by other researchers for fruit byproducts, such as 29 μmol TE/g for blueberry peel.^37^ Collectively, these findings indicate that the antioxidant properties of the samples are substantial and comparable to those of other fruit byproducts.

### SCFAs Production of the Berry Fractions

According to the scientific literature, our findings showed that acetate was the most abundant short-chain fatty acid (SCFA) at 51–71%, followed by propionate at 15–27% and butyrate at 8–21%. The proportions of the principal SCFAs closely matched those reported by Cui et al.^45^ after *in vitro* fermentation of orange pectin, which reported acetate at 67–77%, propionate at 17–26%, and butyrate at 6–11%.

It is noteworthy that the SFF exhibited the highest acetate production (Figure 2), primarily attributable to its soluble fiber content, especially pectin. Both pectin content and acetate production showed a positive correlation (*r* = 0.84, *p* < 0.01) (Figure S2). Additionally, other researchers have reported that acetate production predominantly results from the presence of galacturonic acid and thus from the pectin content.^46^ Furthermore, the PRE and IFF fractions exhibited the second-highest levels of acetate production. Notably, PRE, which serves as a source of EPP, contributed to increased acetate production. These findings are consistent with previous reports involving *in vitro* fermentation of (poly)­phenol extracts from Chilean currants, where acetate levels increased after 24 h of fecal fermentation.^47^ Nonetheless, in the absence of a detailed characterization of the residual fiber matrix and potential coextracted oligosaccharides, this metabolic shift cannot be solely attributed to the (poly)­phenols.^48^

**Figure 2:**
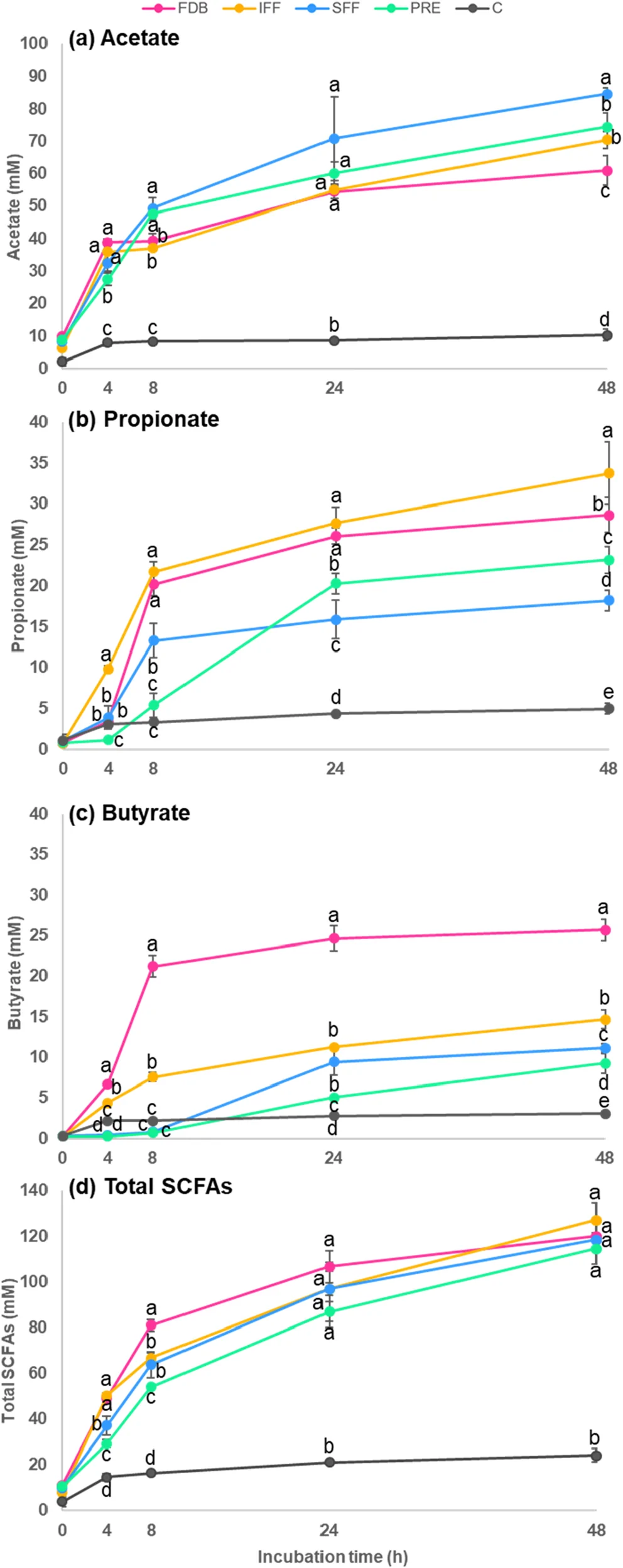
SCFAs production ((a) acetate, (b) propionate, (c) butyrate and (d) total SCFAs) (mM) after 0, 4, 8, 24, and 48 h of in vitro fermentation of freeze-dried berries (Pink circle solid FDB), insoluble fiber-rich fraction (Yellow circle solid IFF), soluble fiber-rich fraction (Blue circle solid SFF), (poly)­phenol extract (Green circle solid PRE) and control (Gray circle solid C). Values are expressed as mean ± SD (*n* = 3). Different letters (a–e) indicate significant differences (*p* < 0.05) among the samples at the same incubation time.

Propionate was predominantly produced in substantial quantities subsequent to the fermentation of IFF and FDB serving as substrates (see Figure 2). Zhao et al.^46^ reported that, following *in vitro* fermentation of pectin and homogalacturonan, the production of propionate exhibits a positive correlation with the content of rhamnose, arabinose, and xylose. These neutral sugars are associated with cellulose and hemicellulose content, which demonstrated higher concentrations in the IFF and FDB fractions compared to SFF (refer to Table 1). Concerning propionate production from the PRE extract, it is noteworthy that it exceeded the production derived from SFF, with EPP and propionate production showing a positive correlation (r = 0.83, *p* < 0.01; see Figure S2). This effect underscores the capacity of the microbiota to metabolize (poly)­phenols from an extract in comparison to a sample with a higher quantity of soluble fiber (SF) and bound (poly)­phenols.

Butyrate production (Figure 2) was highest in FDB, ranging from 0.3 to 25.7 mM. These results may be related to the high carbohydrate content (67.5%) in this fraction, which shows a positive correlation (*r* = 0.86, *p* < 0.01) between the two parameters (Figure S2). This effect may be explained by the fact that one of the metabolic pathways that produces butyrate uses monosaccharides as starting compounds, leading to higher butyrate production compared with the other fractions.^49^

Regarding the total SCFAs content (Figure 2), PRE exhibited total SCFAs production comparable to that of the fiber-rich fractions. Although SCFAs primarily derive from carbohydrate fermentation rather than (poly)­phenols directly, PRE likely coextracted low molecular weight sugars or residual oligosaccharides from the berry matrix. Although the lack of characterization of these carbohydrates prevents us from attributing the observed effect solely to the (poly)­phenols, the results suggest a potential prebiotic-like effect, as previously documented by other authors.^50^ Furthermore, our findings are consistent with those reported by Wu et al.,^51^ who observed increased production of acetate, propionate, and butyrate following *in vitro* incubation of blueberry extract, compared with a control in which no substrate was added.

### Colonic *In Vitro* Biotransformation of (Poly)­phenols in the Berry Fractions

The colonic metabolism of (poly)­phenols during 48 h of fecal fermentation of different fractions obtained from berries (FDB, IFF, SFF, and PRE) was analyzed by HPLC-HR-MS. Table S2 summarizes the 25 (poly)­phenols identified in the fermented samples from the different fractions. A total of 23 (poly)­phenols were quantified during *in vitro* fecal fermentation, as shown in Tables 2–4. These results were obtained using a pooled fecal inoculum, thus reflecting the metabolic activity of a composite microbiota. In general, two main degradation pathways were observed in the fermented samples (Figures S3 and S.4). First, colonic microbiota degraded flavan-3-ols by cleaving the interflavon bond in procyanidins to yield (−)-epicatechin and by producing 5-carbon side-chain ring-fission metabolites (5C-RFMs). Figure S3 shows the proposed metabolic pathway for procyanidin B2 and (−)-epicatechin by Ottaviani et al.,^52^ Borges et al.^53^ and Di Pede et al.^54^ Second, the metabolic pathways for the transformation of ellagic acid into urolithins were observed, as previously described by González-Barrio et al.^55^ and subsequently completed by García-Villalba et al.^56^ (Figure S4).

#### Flavan-3-ols Degradation Pathways and Production of Fecal Microbiota-Derived Metabolites

UHPLC-HRMS analysis of the fermented fecal samples showed the evolution of flavan-3-ols over incubation time and the formation of their microbiota-derived metabolites (Table 2). In this regard, 3 parent flavan-3-ols were identified, namely procyanidin B2, (−)-epicatechin, and (+)-catechin (Table 2). Moreover, eight fecal microbiota-derived flavan-3-ol metabolites were identified, including derivatives of 5C-RFMs (i.e., phenyl-γ-valerolactones and phenylvaleric acids), phenylpropanoic acid, phenylacetic acid, and benzoic acid derivatives.

**Table 2:** Evolution of Flavan-3-ols and Production of Microbiota-Derived Metabolites (μM) in Fecal Slurries after 0, 4, 8, 24, and 48 h of *In Vitro* Fermentation with Berry Fractions[Table-fn t2fn1]

**compounds**	**sample**	**0 h**	**4 h**	**8 h**	**24 h**	**48 h**	***p*** **-value**
procyanidin B2	**FDB**	0.10^bB^	0.21^aB^	0.19^aB^	nd	nd	***
	**PRE**	0.10^bB^	0.10^bB^	0.14^aB^	nd	nd	***
	**IFF**	0.31^bA^	0.52^aA^	0.40^abA^	0.14^cA^	0.14^cA^	***
	**SFF**	0.09 ^aB^	0.10^aB^	nd	nd	nd	***
	*p*-value	***	***	***	***	***	
(−)-epicatechin	**FDB**	2.89^cBC^	16.71^bA^	22.05^aB^	14.26^bB^	3.69^cB^	***
	**PRE**	3.48^bB^	4.51^bB^	11.99^aC^	4.74^bC^	nd	***
	**IFF**	6.86^cA^	19.43^bA^	34.49^aA^	3.69^dC^	nd	***
	**SFF**	2.10^dC^	8.06^bB^	19.53^aB^	20.60^aA^	5.34^cA^	***
	*p*-value	***	***	***	***	***	
(+)-catechin	**FDB**	7.30^aA^	6.37^ab^	3.86^bA^	1.21^cA^	0.79^c^	***
	**PRE**	5.55^aB^	5.27^a^	3.10^bAB^	1.34^cA^	0.62^c^	***
	**IFF**	6.48^aAB^	5.89^a^	2.00^bBC^	1.07^bcA^	0.76^c^	***
	**SFF**	4.03^aC^	4.38^a^	0.86^bC^	0.55^bB^	0.59^b^	***
	*p*-value	***		**	**		
3-hydroxy-3-(3′-hydroxyphenyl)propanoic acid	**FDB**	nd	2.42^bA^	5.21^aA^	nd	nd	***
	**PRE**	nd	0.88^cB^	4.89^aA^	2.47^bB^	nd	***
	**IFF**	nd	2.20^bA^	2.63^aB^	nd	nd	***
	**SFF**	nd	2.63^bA^	5.49^aA^	6.15^aA^	nd	***
	*p*-value		***				
**(R/S**)5-(3′,4′-dihydroxyphenyl)-γ-valerolactone	**FDB**	nd	nd	2.76^aB^	3.03^aB^	0.10^bB^	***
	**PRE**	nd	nd	3.08^aB^	3.67^aC^	1.84^bA^	***
	**IFF**	nd	nd	4.33^bA^	5.62^aA^	0.29^cB^	***
	**SFF**	nd	nd	2.95^bB^	4.90^aA^	5.38^aA^	***
	*p*-value			**	***	***	
**(R/S**)4-hydroxy-5-(3′,4′-dihydroxyphenyl)valeric acid	**FDB**	nd	0.04^cA^	0.10^cA^	0.28^bB^	0.44^aB^	***
	**PRE**	nd	nd	0.04^cB^	0.07^bC^	0.46^aB^	***
	**IFF**	nd	nd	0.09^cA^	0.66^bA^	1.19^aA^	***
	**SFF**	nd	nd	0.04^cB^	0.18^bB^	0.97^aA^	***
	*p*-value		***	**	***	**	
**(R/S**)4-hydroxy-5-(3′-hydroxyphenyl)valeric acid	**FDB**	nd	nd	0.02^b^	0.02^a^	3.29^aB^	***
	**PRE**	nd	nd	0.02^b^	0.02^a^	0.06^aC^	***
	**IFF**	nd	nd	0.02^b^	0.40^a^	11.16^aA^	***
	**SFF**	nd	nd	0.02^b^	0.03^a^	0.23^aC^	***
	*p*-value				***	***	
4-hydroxy-5-(phenyl)valeric acid	**FDB**	nd	0.04^bA^	0.05^bA^	0.05^a^	1.16^aA^	***
	**PRE**	nd	0.02^aB^	0.03^aB^	0.02^b^	0.01^bB^	**
	**IFF**	0.05^bA^	0.05^bA^	0.04^bAB^	0.05^a^	1.21^aA^	***
	**SFF**	0.04^A^	0.03^A^	0.03^AB^	0.04	0.04^B^	
	*p*-value	***	**	*		***	
3-(3′,4′-dihydroxyphenyl)propanoic acid	**FDB**	nd	0.38^ab^	0.44^aBC^	0.22^bcB^	0.16^cB^	**
	**PRE**	nd	0.44^b^	0.66^abAB^	0.71^aA^	0.66^abA^	***
	**IFF**	nd	0.38^b^	0.33^bC^	0.77^aA^	0.22^cB^	***
	**SFF**	nd	0.33^b^	0.82^aA^	0.88^aA^	0.82^aA^	***
	*p*-value			**	***	***	
3-(3′,4′-dihydroxyphenyl)acetic acid	**FDB**	nd	0.42^cB^	0.95^ab^	1.07^aB^	0.71^bC^	***
	**PRE**	nd	0.24^bC^	0.95^a^	1.25^aAB^	1.31^aB^	***
	**IFF**	nd	0.95^cA^	0.95^c^	1.61^bAB^	2.08^aA^	***
	**SFF**	nd	0.42^bB^	0.54^b^	1.84^aA^	1.90^aA^	***
	*p*-value		***		*	***	
3,4-dihydroxybenzoic acid	**FDB**	nd	5.77^bB^	8.05^aA^	5.84^bA^	5.19^bA^	***
	**PRE**	nd	4.22^bcB^	5.64^aB^	4.80^abB^	3.50^cB^	***
	**IFF**	nd	8.95^aA^	7.20^bA^	5.90^cA^	5.00^cA^	***
	**SFF**	nd	2.98^bD^	4.15^aC^	4.09^aB^	3.37^bB^	***
	*p*-value	***	***	***	***	***	

* Values are expressed as mean (*n* = 3). Different lowercase letters indicate significant differences (*p* < 0.05) among the incubation times (0, 4, 8, 24, and 48 h) for the same sample in each row. Different capital letters indicate significant differences (*p* < 0.05) among samples (FDB, PRE, IFF and SFF) at the same incubation time for each compound in each column. Freeze-dried berries (FDB); insoluble fiber-rich fraction (IFF); soluble fiber-rich fraction (SFF); Significant level: ns: not significant. * *p* < 0.05. ** *p* < 0.01. *** *p* < 0.001. nd: not detected.

In the evolution of compound groups shown in Figure 3, the IFF fraction exhibits behavior distinct from the other fractions. It shows a higher release of structurally related (−)-epicatechin compounds at 8 h, followed by nearly complete metabolization by 24 h of fermentation. This observation suggests that the type of DF influences the rate of metabolization, with IF delaying the process. This delay leads to greater metabolic activity at later fermentation times, resulting in increased phenylvaleric acid derivatives at 48 h—a phenomenon not observed in any other fraction.

**Figure 3:**
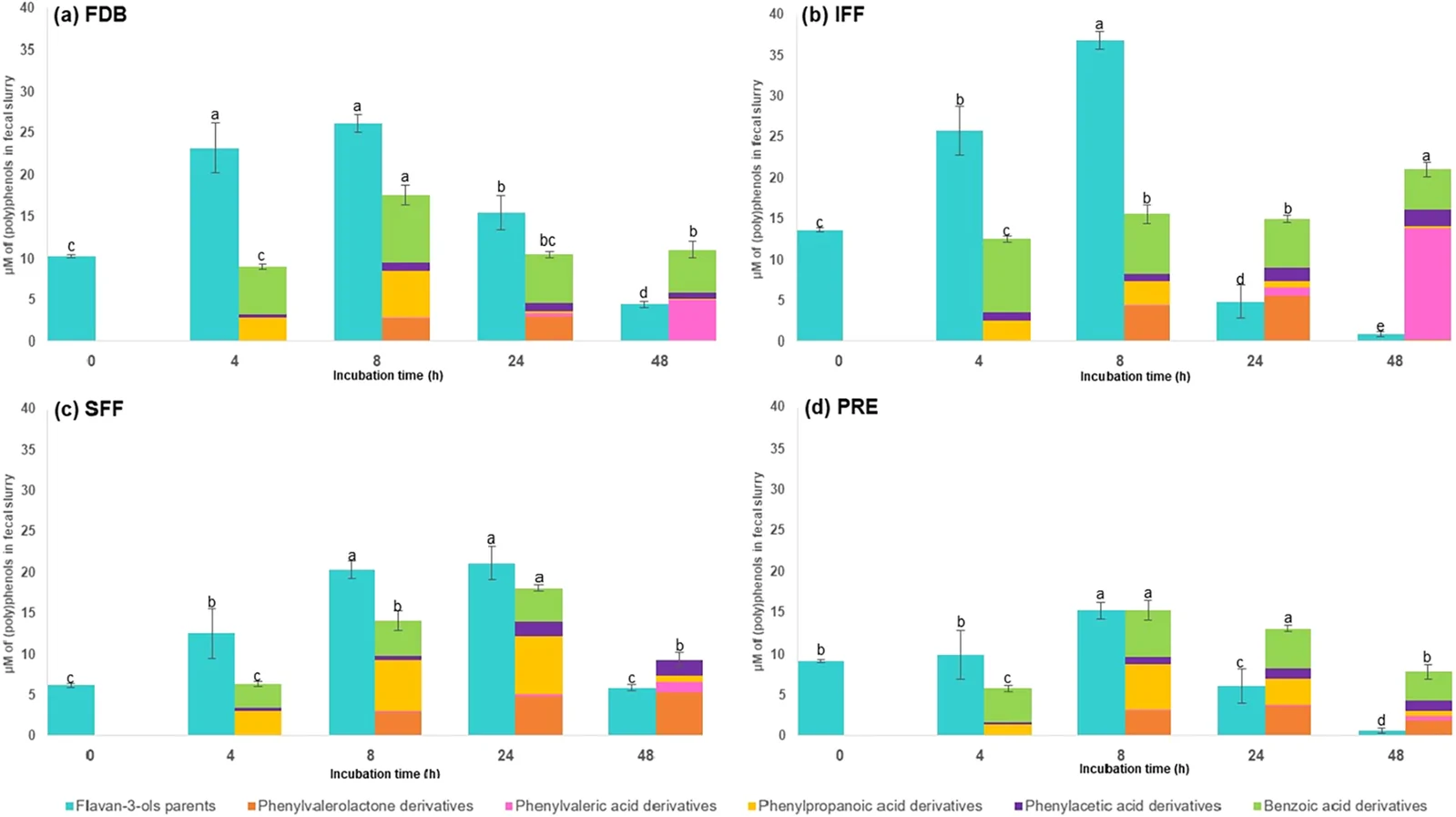
Total flavan-3-ol parents (Blue box solid) and total metabolites (μM), calculated as the sum of phenylvalerolactone derivatives (Brown box solid), phenylvaleric acid derivatives (Pink box solid), phenylpropanoic acid derivatives (Yellow box solid), phenylacetic acid derivatives (Purple box solid), and benzoic acid derivatives (Green box solid), for freeze-dried berries (FDB (a)), insoluble fiber fraction (IFF (b)), soluble fiber fraction (SFF (c)), and (poly)­phenol-rich extract (PRE (d)). Different letters (a–d) indicate significant differences (p *<* 0.05) among fermentation times (0, 4, 8, 24, and 48 h) for flavan-3-ol parents and total metabolites.

Flavan-3-ol monomers, mainly (−)-epicatechin and (+)­catechin, were found at the highest levels at the start of fermentation. These compounds have previously been described as the main components of red fruits, such as strawberry, blackcurrant, and blueberry.^57–59^

The evolution of parent flavan-3-ols during fermentation varied among fractions (Table 2). Procyanidin B2 was present at higher levels in the IFF fraction than in the others. Its concentration increased significantly in the FDB and IFF fractions at 4 h, while it remained unchanged in PRE and SFF. However, it dropped to undetectable levels after 24 h in most fractions, except in IFF. Previous research has observed that most procyanidin content in grape seeds is completely metabolized after 10 h of fecal fermentation.^60^ For the IFF fraction, a basal concentration of 0.14 μM remained after 48 h of fermentation (Table 2). This may be because procyanidin B2 is bound to IF and is continuously released throughout fecal fermentation. In this sense, IFF showed the highest IF content (Table 1). Furthermore, previous research has reported a high affinity of IF for procyanidins.^61^ (−)-Epicatechin also showed the highest content in the IFF fraction at 0 h. All fractions increased significantly after 8 h of fermentation, especially in IFF and FDB. This increase may be due to cleavage of interflavonoid bonds, yielding (−)-epicatechin monomers, as well as the release of bound phenolics.^53^ Subsequently, (−)-epicatechin decreased in FDB, PRE, and IFF fractions after 24 h, while SFF decreased after 48 h of fecal fermentation. On the other hand, (+)-catechin was higher in FDB and IFF fractions than in SFF prior to fermentation (Table 2). The content of this compound was stable after 4 h and significantly decreased after 8 h in all fractions.

3-Hydroxy-3-(3′-hydroxyphenyl)­propanoic acid was found at 4 h of incubation, mainly in FDB, IFF and SFF fractions. This is formed by the fragmentation of the heterocyclic ring (C-ring) of (−)-epicatechin and the loss of the hydroxyl in the 4’-position, keeping a phenol ring (B-ring) and bound to a propanoic acid (Figure S3). This metabolite was identified as a radiolabeled (−)-epicatechin degradation product in previous research.^52^ Notably, its concentration significantly increased at 8 h, decreasing after 24 h in all fractions, except for SFF. Furthermore, this compound was not observed at 48 h in any of the fractions. Borges et al.^53^ suggest that the formation of 3-hydroxy-3-(3′-hydroxyphenyl)­propanoic acid derived from (−)-epicatechin is formed in an alternative 5C-RFMs pathway, being its metabolic origin currently uncertain.^62^

On the contrary, (−)-epicatechin can be transformed from a C6–C3–C6 structure to a C6–C9 structure by opening the C-ring and breaking the O1–C2 bond, forming an intermediate metabolite called 5-(3′,4’-dihydroxyphenyl)-3-(2”,4”,6”-trihydroxyphenyl)­propan-2-ol, which is unstable and was not found in our samples. Afterward, this intermediate loses an oxaloacetate group, forming (R/S)­5-(3′,4’-dihydroxyphenyl)-γ-valerolactone, a C6–C5 5C-RFCs (Figure S3). This compound was detected at quantifiable concentrations at 8 h, with concentrations in the IFF and SFF fractions increasing after 24 h (Table 2). Through fecal microbiota activity, (R/S)­4-hydroxy-5-(3′,4’-dihydroxyphenyl)­valeric acid is formed from (R/S)­5-(3′,4’-dihydroxyphenyl)-γ-valerolactone after hydroxylation of the valerolactone ring (Figure S3). This compound was found at low concentrations after 4 h in the FDB fraction and after 8 h in the rest. Subsequently, this metabolite increased significantly after 48 h, especially in IFF, reaching the highest concentration (1.19 μM), probably related to its DF content (Table 1).

(R/S)­4-hydroxy-5-(3′-hydroxyphenyl)­valeric acid was quantified after 8 h of fermentation, significantly increasing at 48 h (Table 2). Especially, IFF fraction showed the highest concentration after 48 h (11.16 μM). This compound is formed directly by dehydroxylation of (R/S)­4-hydroxy-5-(3′,4’-dihydroxyphenyl)­valeric acid as described by Di Pede et al.^54^ In this sense, 4-Hydroxy-5-(phenyl)­valeric acid was observed at a very low level throughout the fecal fermentation in all fractions. This metabolite results from the dehydroxylation of (R/S)­4-hydroxy-5-(3′-hydroxyphenyl)­valeric acid (Figure S3). FDB and IFF showed a significant increase of this compound after 48 h, reaching 1.16 and 1.21 μM, respectively. This fact seems to indicate that the transformation of the (poly)­phenols of IFF fraction was slower than the rest, maybe due to its fiber content, that probably delayed the release of compounds, and consequently the (poly)­phenol metabolism (Tables 1 and 2). In addition to the formation of 4-hydroxy-5-(phenyl)­valeric acid derivatives, phenyl-γ-valerolactones may also undergo alternative metabolic transformations leading to 5-(phenyl)­valeric acids, as reported in the literature. This indicates the existence of parallel metabolic routes during flavan-3-ol catabolism.

3-(3′,4′-Dihydroxyphenyl)­propanoic acid was detected at low concentrations in all fractions starting at 4 h, with concentrations increasing at 8 h in the FDB, PRE, and SFF fractions and at 24 h in the IFF fraction. The content of this compound in PRE and SFF remained at 48 h (Table 2). This metabolite has been reported to be one of the most abundant microbial metabolites derived from the opening of the upper unit of procyanidin.^54,60^

On the one hand, 3-(3′,4’-dihydroxyphenyl)­acetic acid was first quantified after 4 h of fecal fermentation across different fractions. FDB and PRE showed similar trends, increasing their content at 8 h and remaining stable thereafter. However, the concentration of this compound began to decline in FDB, whereas PRE remained stable. On the other hand, a gradual increase was observed in IFF and SFF after 24 h, due to the release of compounds bound to DF molecules, reaching the highest concentration at 48 h (Table 2). This compound is obtained from the α-oxidation of 3-(3′,4’-dihydroxyphenyl)­propanoic acid.^60^ Moreover, the transformation of the upper unit may involve C-ring cleavage followed by side-chain shortening reactions, ultimately yielding 3′,4′-dihydroxyphenylacetic acid from epicatechin-derived intermediates.

Similarly, 3,4-dihydroxybenzoic acid was detected at 4 h. This may be due to its formation via the α-oxidation of 3′,4’-dihydroxyphenyl)­acetic acid, the dehydroxylation of 3,4,5-trihydroxybenzoic acid, and the microbial cleavage of the top unit of dimeric procyanidins, as shown in Figure S3.^63^ The content of this compound increased significantly at 4 and 8 h, then decreased gradually to 48 h. The content of 3,4-dihydroxybenzoic acid in the different fractions remained high, ranging from 3.37 μM in SFF to 5.19 μM in FDB. The IFF fraction reached the highest concentration of this compound, followed by FDB. Thus, IFF reached the highest content after 8 h, while FDB peaked at 4 h (Table 2). These results showed that the IFF fraction reached the highest amounts, but the IF affected the transformation of (poly)­phenols during fermentation, making it slower than in the other fractions. Although some phenolic acids are commonly included within flavan-3-ol degradation pathways, such as 3′,4’-dihydroxyphenylacetic acid or 3,4-dihydroxybenzoic acid, they may also originate from the microbial metabolism of other phenolic families, which may contribute to variations in their observed levels.

The evolution of (−)-epicatechin-derived metabolite groups for each fraction is presented in Figure S5 of the Supporting Information. These graphs show the temporal changes in structurally related (−)-epicatechin metabolites, phenyl-γ-valerolactones, phenylvaleric acid derivatives and phenolic acids during fermentation.

#### Ellagic Acid Degradation Pathways and Production of Fecal Microbiota-Derived Metabolites

UHPLC-HRMS analysis of the fermented fecal samples showed degradation of ellagic acid-related compounds by the fecal microbiota over the incubation period and the formation of urolithins, which are gut microbiota-derived metabolites (Figure S4). Four native ellagic acid parents and ellagitannins were identified at the beginning of the incubation, including ellagic acid, ellagic acid hexoside, ellagic acid deoxyhexoside, and valoneic acid dilactone (Table 3). In addition, 6 microbiota-derived ellagic acid metabolites were produced, including 3,4,8,9,10-pentahydroxy-urolithin (urolithin M5), 3,4,8,9-tetrahydroxy-urolithin (urolithin D), 3,8,9,10-tetrahydroxy-urolithin (urolithin M6), 3,8,9-trihydroxy-urolithin (urolithin C), 3,8,10-trihydroxy-urolithin (urolithin M7) and, 3,8-dihydroxy-urolithin (urolithin A).

**Table 3:** Transformation of Ellagic Acids and Production of Microbiota-Derived Metabolites (μM) in Fecal Slurries after 0, 4, 8, 24, and 48 h of *in Vitro* Fermentation with Berry Fractions[Table-fn t3fn1]

compounds	**sample**	**0**	**4**	**8**	**24**	**48**	*p*-value
ellagic acid	**FDB**	6.44^aB^	4.79^bB^	4.58^bB^	1.02^cB^	0.84^cB^	***
	**PRE**	3.07^aC^	2.02^bC^	1.60^bC^	0.90^cB^	0.57^cB^	***
	**IFF**	11.93^aA^	10.33^bA^	7.29^cA^	1.23^dB^	0.96^dB^	***
	**SFF**	3.94^bC^	5.12^aB^	5.09^aB^	5.51^aA^	5.27^aA^	**
	*p-*value	***	***	***	***	***	
ellagic acid hexoside	**FDB**	0.97^aB^	0.22^b^	0.11^cA^	nd	nd	***
	**PRE**	0.30^aC^	0.19^b^	nd	nd	nd	***
	**IFF**	1.51^aA^	0.15^b^	0.11^bA^	nd	nd	***
	**SFF**	0.34^aC^	0.15^b^	nd	nd	nd	***
	*p*-value	***		***			
ellagic acid deoxyhexose	**FDB**	0.27^aA^	0.18^bA^	0.13^cB^	nd	nd	***
	**PRE**	0.22^aB^	0.18^bA^	0.16^cA^	nd	nd	***
	**IFF**	0.27^aA^	0.18^bA^	0.13^cB^	0.11^cA^	nd	***
	**SFF**	0.16^aC^	0.13^bB^	0.13^cB^	nd	nd	***
	*p*-value	***	**	***	***		
valoneic acid dilactone	**FDB**	0.19^bB^	0.29^aC^	0.29^aC^	0.19^bBC^	0.17^bB^	***
	**PRE**	0.19^cB^	0.30^aC^	0.26^bC^	0.17^cdC^	0.15^dC^	***
	**IFF**	0.25^bA^	0.44^aA^	0.44^aA^	0.21^bcB^	0.18^cB^	***
	**SFF**	0.26^bcA^	0.36^aB^	0.37^aB^	0.27^bA^	0.21^cA^	***
	*p*-value	***	***	***	***	***	
3,4,8,9,10-pentahydroxy-urolithin (urolithin M5)	**FDB**	nd	1.18^bB^	4.63^aB^	0.23^cC^	0.14^cB^	***
	**PRE**	nd	0.51^bC^	0.76^aC^	0.90^aB^	0.37^bB^	***
	**IFF**	nd	11.31^bA^	12.23^aA^	0.41^cC^	0.31^cB^	***
	**SFF**	nd	1.20^cB^	1.62^cC^	2.46^bA^	4.98^aA^	***
	*p*-value		***	***	***	***	
3,4,8,9-tetrahydroxy-urolithin (urolithin D)	**FDB**	nd	nd	0.10^bAB^	0.13^aB^	0.10^bB^	***
	**PRE**	nd	nd	0.08^bAB^	0.09^bB^	0.11^aB^	***
	**IFF**	nd	0.11^bA^	0.13^bA^	0.48^aA^	0.54^aA^	***
	**SFF**	nd	nd	0.05^bB^	0.09^abB^	0.13^aB^	***
	*p*-value		***	*	***	***	
3,8,9,10-tetrahydroxy-urolithin (urolithin M6)	**FDB**	nd	2.61^bB^	4.27^aB^	0.63^cB^	0.27^cC^	***
	**PRE**	nd	0.17^bC^	0.24^bC^	2.01^aA^	1.77^aB^	***
	**IFF**	nd	6.32^bA^	12.51^aA^	0.58^cB^	0.20^cC^	***
	**SFF**	nd	0.33^cC^	0.63^bC^	0.82^bB^	2.15^aA^	***
	*p*-value		***	***	***	***	
3,8,9-trihydroxy-urolithin (urolithin C)	**FDB**	nd	nd	0.11^cA^	2.13^aAB^	0.47^bB^	***
	**PRE**	nd	nd	nd	0.14^bB^	0.28^aB^	***
	**IFF**	nd	nd	0.13^bA^	3.94^aA^	1.65^bA^	***
	**SFF**	nd	nd	0.09^bB^	0.13^bB^	0.26^aB^	***
	*p*-value			***	**	***	
3,8,10-trihydroxy-urolithin (urolithin M7)	**FDB**	nd	nd	nd	0.16^aB^	0.17^aB^	***
	**PRE**	nd	nd	nd	0.09^bC^	0.11^aC^	***
	**IFF**	nd	nd	nd	0.24^aA^	0.27^aA^	***
	**SFF**	nd	nd	nd	0.09^aC^	0.09^aC^	***
	*p*-value				***	***	
3,8-dihydroxy-urolithin (urolithin A)	**FDB**	nd	nd	nd	3.01^aB^	3.33^aB^	***
	**PRE**	nd	nd	nd	0.51^aC^	0.39^aC^	***
	**IFF**	nd	nd	nd	4.55^bA^	5.77^aA^	***
	**SFF**	nd	nd	0.36^bA^	0.54^aC^	0.39^bC^	***
	*p*-value			***	***	***	

* Values are expressed as mean (*n* = 3). Different lowercase letters indicate significant differences (*p* < 0.05) among the incubation times (0, 4, 8, 24, and 48 h) for the same sample in each row. Different capital letters indicate significant differences (*p* < 0.05) among samples (FDB, PRE, IFF and SFF) at the same incubation time for each compound in each column. Freeze-dried berries (FDB); insoluble fiber-rich fraction (IFF); soluble fiber-rich fraction (SFF); Significant level: ns: not significant. * *p* < 0.05. ** *p* < 0.01. *** < 0.001. nd: not detected.

Across the evolution of compound groups shown in Figure 4, the IFF fraction exhibited the most notable differences, as seen in the flavan-3-ols group, again suggesting that the type of DF influences the metabolic pathways of the present compounds. In the IFF fraction, the native ellagic acid and ellagitannins group undergoes gradual degradation, likely due to progressive release from the matrix, leading to increased production of penta- and tetrahydroxy-urolithins at 8 h of fermentation and of tri- and dihydroxy-urolithins at 24 h. This effect was not observed in the SFF fraction, where native ellagic acid and ellagitannins compounds remained stable throughout fermentation, suggesting that the fiber’s properties—potentially related to water retention and gel-forming capacity—reduced the bioaccessibility of these compounds.

**Figure 4:**
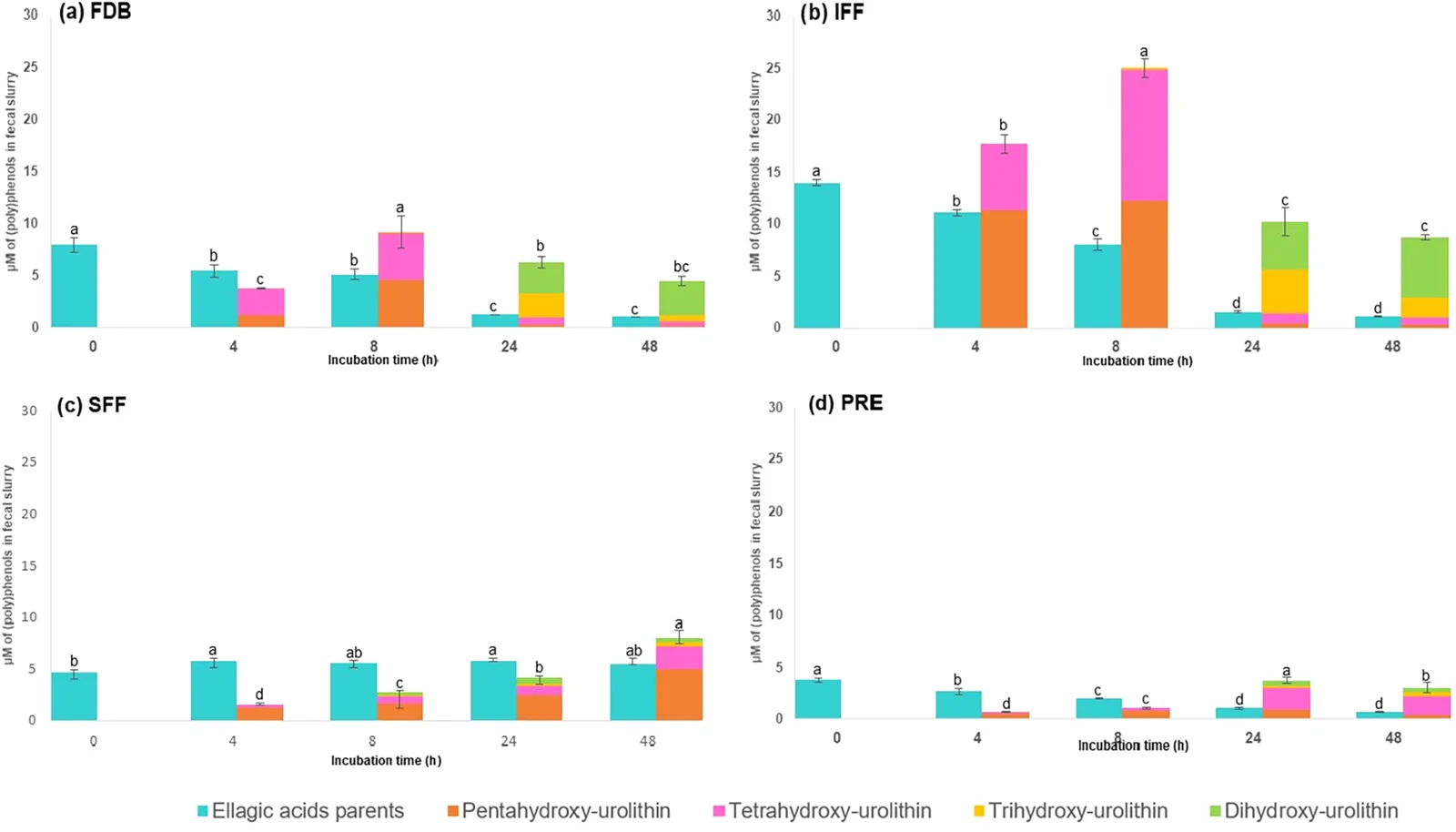
Total native ellagic acid and ellagitannins (Blue box solid) and total metabolites (μM), calculated as the sum of pentahydroxy-urolithin (Brown box solid), tetrahydroxy-urolithin (Pink box solid), trihydroxy-urolithin (Yellow box solid), and dihydroxy-urolithin (Green box solid), for freeze-dried berries (FDB (a)), insoluble fiber fraction (IFF (b)), soluble fiber fraction (SFF (c)), and (poly)­phenol-rich extract (PRE (d)). Different letters (a–d) indicate significant differences (p *<* 0.05) among fermentation times (0, 4, 8, 24, and 48 h) for native ellagic acid and ellagitannins and total metabolites.

Regarding native ellagic acid and ellagitannins, it was observed that these compounds was gradually degraded during fermentation, as previously reported.^23^ It was noted that ellagic acid was significantly higher in the IFF fraction, followed by FDB (Table 3). These results indicate a higher content of NEPP (Figure 1 and Table 3), due to the linkage of (poly)­phenols with the heteropolysaccharides that constitute these fibers.^64^ This compound showed gradual degradation throughout fecal fermentation of the FDB, PRE, and IFF fractions, with significantly lower levels at 48 h. Conversely, the SFF fraction showed a different behavior, increasing after 4 h and remaining stable until the end of fermentation. This behavior could be due to the higher swelling capacity associated with the pectin content (gel-forming fibers), as well as the nature of the (poly)­phenol-polysaccharide interaction,^65^ with ellagic acid progressively released from the soluble fiber matrix and transformed during fecal fermentation (Table 3).

A hexoside of ellagic acid was also quantified at higher concentrations in the IFF fraction. The content of this compound decreased during fermentation and was not detected after 24 h in any fractions. Likewise, ellagic acid deoxyhexose decreased significantly after 8 h in all fractions and was undetectable at 24 h, except in the IFF fraction. Valoneic acid dilactone was also observed at the beginning of fermentation. This compound increased significantly after 4 and 8 h, especially in the IFF and SFF fractions. Some authors have previously observed that valoneic acid dilactone is bound to solid and nonextractable components and must be released by hydrolysis.^66^

3,4,8,9,10-Pentahydroxy-urolithin (urolithin M5) was detected from 4 h onward in different fractions. This compound forms via lactone ring opening of ellagic acid followed by decarboxylation.^56^ The IFF fraction had the highest concentration of this compound, 10-fold higher than in the other fractions, and this correlated directly with the amount of parent ellagic acid (Table 3). The content continued to increase significantly after 8 h in the FDB, PRE, and IFF fractions, whereas no significant changes were observed in the SFF fraction at this time point. After 24 h, the levels of urolithin M5 decreased in the FDB and IFF fractions. Conversely, the SFF fraction showed an opposite trend, with a continued increase across stages and reaching the highest concentration after 48 h. The delayed formation of 3,4,8,9,10-pentahydroxy-urolithin (urolithin M5) in the SFF fraction appears to be directly related to the sustained release of nonextractable ellagic acid (see Figure 1b and Table 3).

From 3,4,8,9,10-pentahydroxy-urolithin (urolithin M5), two derived molecules were detected in the analyzed samples, namely 3,4,8,9-tetrahydroxy-urolithin (urolithin D) (loss of hydroxyl at position 10) and 3,8,9,10-tetrahydroxy-urolithin (urolithin M6) (loss of hydroxyl at position 4). Regarding 3,4,8,9-tetrahydroxy-urolithin (urolithin D), this biomolecule was observed only in the IFF fraction after 4 h, while it was found in all fractions after 8 h. The IFF fraction maintained the highest concentrations in the later stages (24 and 48 h), and it showed the highest levels at these stages compared with the rest (Table 3). For the remaining fractions, the highest levels in each were reached after 24 h for FDB fractions and after 48 h for PRE and SFF fractions (Table 3). Conversely, the evolution of 3,8,9,10-tetrahydroxy-urolithin (urolithin M6) differed among the evaluated fractions. Thus, FDB and IFF reached the highest content after 8 h, with 4.27 and 12.51 μM, respectively. PRE and SFF reached their highest levels at later stages, but with lower concentrations of 3,8,9,10-tetrahydroxy-urolithin (urolithin M6) than FDB or IFF fractions. In general, the content of 3,8,9,10-tetrahydroxy-urolithin (urolithin M6) in different fractions was higher than that of 3,4,8,9-tetrahydroxy-urolithin (urolithin D). It is noted that 3,4,8,10-tetrahydroxy-urolithin (urolithin E), which can be formed from 3,4,8,9,10-pentahydroxy-urolithin (urolithin M5) by dehydroxylation of the hydroxyl at position 9, was not found in the analyzed samples. According to the literature, this catabolite was quantified at lower concentrations than 3,8,9,10-tetrahydroxy-urolithin (urolithin M6) and 3,4,8,9-tetrahydroxy-urolithin (urolithin D) in fecal fermentation experiments, and its production is highly dependent on specific gut bacterial strains.^67,68^

3,8,9-Trihydroxy-urolithin (urolithin C) was the primary trihydroxy urolithin observed after 8 h of fecal fermentation across most fractions. This biomolecule can be formed from 3,4,8,9-tetrahydroxy-urolithin (urolithin D) by loss of the hydroxyl at position 4, or from 3,8,9,10-tetrahydroxy-urolithin (urolithin M6) by loss of the hydroxyl at position 1 (Figure S4). This compound reached its highest concentration after 24 h in the FDB and IFF fractions, whereas the PRE and SFF fractions reached it after 48 h. Notably, the IFF fractions reached the highest concentration after 24 h (3.94 μM), primarily derived from the fragmentation of urolithin M6.

3,8,10-Trihydroxy-urolithin (urolithin M7) was another trihydroxy urolithin detected in fecal samples. This compound is derived from 3,8,9,10-tetrahydroxy-urolithin (urolithin M6) by cleavage of the hydroxyl group at position 9. Urolithin M7 was observed during the later stages of fecal fermentation, primarily in the IFF and FDB fractions (Table 3).

A dihydroxy urolithin was identified in the samples, namely 3,8-dihydroxy-urolithin (urolithin A). This can be formed from urolithin C or M7 by loss of the hydroxyl group at position 9 or 10, respectively (Figure S4). The IFF fraction showed the highest content of this compound, especially after 48 h (Table 3). This compound has been associated with many health effects in consumers, such as cellular and mitochondrial health, anti-inflammatory effects, and effects on aging, among others.^7^ It is noted that 3-hydroxy-urolithin (urolithin B) was not found in any sample. This is probably because the volunteers belong to the urolithin metabotype A group, which mainly produces urolithin A derivatives.^69^ In this respect, some authors have observed that the different urolithin metabotypes (A, B, or 0) among people are highly dependent on the gut microbiota composition.^67^

The evolution of ellagic acid-derived metabolites for each fraction is presented in Figure S6 of the Supporting Information. These graphs show the temporal changes in parent ellagic acids and the sequential formation of urolithin derivatives across fermentation time for each fraction.

#### Gallic Acid Derived Compounds

Additionally, other phenolic acids were detected in fecal samples. A high concentration of gallic acid was observed throughout all stages of fermentation. FDB, PRE, and SFF showed a similar trend, with concentrations increasing at the start of incubation and reaching their highest levels after 8 h (Table 4). The evolution of gallic acid in the IFF fraction was slightly different, with the highest concentration after 4 h of fermentation. The content of this compound remained high throughout the different fermentation times, compared with the previously identified compounds. This compound can be released from phenolic polymers, such as proanthocyanidins, gallotannins, and ellagitannins.^70^ Additionally, a gallic acid hexoside was observed in the early stages of fermentation, especially in the FDB fraction. Gallic acid (3,4,5-trihydroxybenzoic acid) is primarily metabolized by gut microbiota through decarboxylation and dehydroxylation reactions to the formation of simpler phenolic compounds, including pyrogallol, catechol and other low-molecular-weight benzene derivatives. Such pathways are consistent with the general microbial degradation of dietary (poly)­phenols, involving progressive loss of hydroxyl groups and aromatic simplification.^71^

**Table 4:** Gallic Acid Derivatives (μM) Detected in Fecal Slurries after 0, 4, 8, 24, and 48 h of *In Vitro* Fermentation with Berry Fractions[Table-fn t4fn1]

compounds	**sample**	**0**	**4**	**8**	**24**	**48**	*p*-value
3,4,5-trihydroxybenzoic acid (gallic acid)	**FDB**	2.65^dC^	38.62^bB^	55.78^aA^	24.98^cA^	4.06^dB^	***
	**PRE**	2.82^dC^	21.40^bC^	33.09^aB^	14.58^cB^	8.11^cdA^	***
	**IFF**	12.70^cA^	92.58^aA^	60.02^bA^	9.17^cB^	8.17^cA^	***
	**SFF**	7.99^dB^	18.87^bC^	22.63^aB^	11.76^cB^	5.06^dAB^	***
	*p*-value	***	***	***	***	**	
3,4,5-trihydroxybenzoic acid glucose (monogalloyl glucose)	**FDB**	2.86^aA^	2.53^bB^	nd	nd	nd	***
	**PRE**	1.87^aB^	3.35^aA^	1.18^bA^	nd	nd	***
	**IFF**	1.60^aB^	nd	nd	nd	nd	***
	**SFF**	1.57^aB^	1.94^bB^	nd	nd	nd	***
	*p*-value	***	***	***			

* Values are expressed as mean (*n* = 3). Different lowercase letters indicate significant differences (*p* < 0.05) among the incubation times (0, 4, 8, 24, and 48 h) for the same sample in each row. Different capital letters indicate significant differences (*p* < 0.05) among samples (FDB, PRE, IFF and SFF) at the same incubation time for each compound in each column. Freeze-dried berries (FDB); insoluble fiber-rich fraction (IFF); soluble fiber-rich fraction (SFF); Significant level: ns: not significant. * *p* < 0.05. ** < 0.01. *** < 0.001. nd: not detected.

In conclusion, the extraction of both fiber fractions from the berry’s mixture (IFF and SFF) yielded two distinct products with differing fiber compositions: one with a high content of IF and the other with SF. Both fractions are rich in NEPP, predominantly ellagic acid. Furthermore, the different fiber compositions influenced the metabolism of the (poly)­phenols presented, with IFF exhibiting a more gradual metabolism throughout fermentation due to its highest fiber content and specific fiber characteristics. Notably, IFF also exhibited a greater production of metabolites derived from flavan-3-ols and ellagic acid, highlighting the impact of its fiber composition on microbial metabolism. Additionally, a (poly)­phenol extract (PRE) demonstrated a potentially prebiotic effect comparable to that of a fiber-rich matrix in terms of SCFAs production. These results present an intriguing scenario, as the design of a prebiotic ingredient rich in bioactive compounds must consider both the fiber composition and the interactions between these compounds to fully understand their potential health benefits.

Although this study provides valuable insights into the interactions between berry-derived fiber fractions and (poly)­phenol metabolism under controlled *in vitro* conditions, several limitations should be acknowledged. First, the findings cannot be directly extrapolated to human digestion, where complex dietary matrices, meal composition, and gastrointestinal transit times introduce additional variables that may alter bioaccessibility and microbial transformation patterns. Second, the substantial interindividual variability in human fecal microbiota composition—particularly in urolithin metabotypes and SCFAs-producing capacity—means that the metabolite profiles observed here may not be representative of diverse populations. In addition, fecal samples from multiple donors were pooled to obtain a single composite inoculum, thereby removing interindividual variability and potentially influencing microbial dynamics. Consequently, the results reflect a composite microbiota rather than individual-specific responses and may differ from those obtained using individual donors. Additionally, since microbiota composition was not directly analyzed and only SCFAs production was evaluated, a prebiotic effect cannot be definitively established and should therefore be considered potential. Finally, while the pooled inoculum from healthy women provides a standardized model, it does not capture the full spectrum of microbiota diversity encountered in real-world scenarios. Future *in vivo* studies are needed to validate these findings and assess their physiological relevance.

## Supplementary Material



## Notes

Detailed experimental procedures for the in vitro digestion and fecal fermentation, preparation of freeze-dried berries, insoluble and soluble fiber-rich fractions, and (poly)­phenol-rich extracts; assessments of antioxidant capacity; correlation analyses between short-chain fatty acid production and berry composition parameters; tentative identification of (poly)­phenols and their microbiota-derived metabolites from in vitro fecal fermentation; and proposed degradation pathways for procyanidin B2 and ellagic acid (PDF)

## Abbreviations

ANOVA: analysis of variance
DF: dietary fiber
EPP: extractable polyphenols
FDB: freeze-dried berries
FRAP: ferric reducing antioxidant power
GC-FID: gas chromatography–flame ionization detector
GLC-FID: gas–liquid chromatography with flame ionization detector
HPLC: high-performance liquid chromatography
HPLC-HR-MS: high-performance liquid chromatography–high-resolution mass spectrometry
IF: insoluble fiber
IFF: insoluble fiber-rich fraction
INFOGEST: standardized *in vitro* gastrointestinal digestion protocol
MSIMI: Metabolite Standards Initiative Metabolite Identification Level
NEPP: nonextractable polyphenols
ORAC: oxygen radical antioxidant capacity
PRE: (poly)­phenol-rich extract
SCFAs: short-chain fatty acids
SF: soluble fiber
SFF: soluble fiber-rich fraction
SWC: swelling capacity
TE: Trolox equivalents
TF: total fiber
UHPLC-HRMS: ultrahigh-performance liquid chromatography–high-resolution mass spectrometry
USDA: United States Department of Agriculture
WRC: water retention capacity
5C-RFMs: five carbon side chain ring fission metabolites
5C-RFCs: five carbon ring fission compounds
