# Supplementary material for: Bioaccessibility and Microbial Metabolism of (Poly)phenol-Rich Dietary Fiber Fractions from Frozen Mixed Berries in an In Vitro Model
Source: J Agric Food Chem. 2026 Oct 21;74(43):33805–19. doi: 10.1021/acs.jafc.6c01779 (PMC13741838; doi:10.1021/acs.jafc.6c01779)
Supplement: Supplementary file 1 [file jf6c01779_si_001.pdf]

SUPPORTING INFORMATION

Bioaccessibility and microbial metabolism of (poly)phenol-rich dietary fiber fractions from frozen mixed berries in an in vitro model

Vanesa Núñez-Gómez[ab#], José Luis Ordóñez-Díaz[c#], Jose Manuel Moreno-Rojas[c,d], Gema Pereira-Caro[c,d], María Jesús Periago[a,b], Rocío González-Barrio[a,b]*

[#]Contributed equally to this article.

**Corresponding autor. E-mail address: rgbarrio@um.es (R. González-Barrio).*

[a]Department of Food Technology, Food Science and Nutrition, University of Murcia. Campus Mare Nostrum, Campus de Espinardo, 30100 Murcia, Spain.

[b]Biomedical Research Institute of Murcia Pascual Parrilla–IMIB, 30120 Murcia, Spain

[c]Area of Agroindustry and Food Quality, Andalusian Institute of Agricultural and Fisheries Research and Training (IFAPA), Alameda del Obispo, Avda. Menéndez-Pidal, 14004 Córdoba, Spain.

[d]Foods for Health Group, Instituto Maimónides de Investigación Biomédica de Córdoba (IMIBIC), 14004 Córdoba, Spain.

Extended version for the in vitro digestion and colonic fermentation

***In vitro* digestion.** Simulated gastrointestinal digestion was carried out following the *in vitro* method from INFOGEST protocol (Brodkorb *et al*., 2019). To start the process with the oral phase, 0.5 g of each FDB, IFF, SFF, and PRE were mixed in individual containers with simulated salivary fluid, which includes α-amylase. The sample was stirred for 2 min at 37 °C, to simulate mastication. The gastric phase was then carried out using simulated gastric fluid, which includes pepsin, the sample was adjusted to pH 3 and was placed in a water bath with stirring for 2 h at 37 °C. The last step was the intestinal phase, in this case simulated intestinal fluid, which includes bile salts and pancreatin was used, the mixture was adjusted to pH 7 and then placed in a water bath with stirring for 2 h at 37 °C. Finally, to slow down the enzymatic activity, the samples were stored at -80 °C and then they were freeze-dried and kept on cold until the *in vitro* fermentation process was carried out.

***In vitro* fecal fermentation.** Fecal samples were collected from nine healthy, normal-weight women aged 25–35 years. Inclusion criteria were: non-smoking status, stable dietary habits, absence of gastrointestinal disease symptoms, no antibiotic use within 4–6 months prior to the study, no adherence to dietary restrictions, and no use of food supplements, prebiotics, or probiotics. Moreover, the participants had to follow a pre-established diet for two days prior to the study, mainly this diet removed the main sources of fibre and (poly)phenols. Fresh faeces were collected in a flask containing an AnaeroGenTM Sacket (AN35, Oxoid®, UK), to produce anaerobic conditions and avoid microbial modifications. The samples were processed in the following 2 h.

The fermentation medium was prepared as follows: 2 g of tryptone were dissolved in 400 mL of distilled water and 100 μL of micromineral solution (consisting of 13.2 g of $CaCl_2 \cdot 2H_2O$, 10 g

of $MnCl_2 \cdot 4H_2O$, 1 g of $CoCl_2 \cdot 6H_2O$, 8 g of $FeCl_3 \cdot 6H_2O$, and distilled water up to 100 mL), 200 mL of buffer (2 g of NH_4HCO_3, 17.5 g of $NaHCO_3$, and distilled water up to 500 mL), 200 mL of macromineral solution (2.85 g of Na_2HPO_4, 3.1 g of KH_2PO_4, 0.3 g of $MgSO_4 \cdot 7H_2O$, and distilled water up to 500 mL), and 1 mL of 1% of resazurin solution (w/v), which was used as a redox indicator, were added. Then, the medium was adjusted to pH 7 using 6 M HCl and then sterilized before allowing cooling under oxygen free nitrogen for 30 min, the medium changed from blue to pink when all the oxygen was removed. Reducing solution (312 mg of cysteine hydrochloride, 2 mL of 1 M NaOH, 312 mg of $Na_2S \cdot 9H_2O$, and distilled water up to 50 mL) was added in a proportion of 1 mL of reducing solution per 20 mL of medium, after which the solution was purged with nitrogen until anaerobic conditions.

Samples of fresh feces were homogenized with 0.07 M sodium phosphate buffer to obtain 32% fecal suspensions. Five mL of fecal suspension was added to 44 mL of fermentation medium at pH 7 and placed in a 100 mL McCartney bottle. Freeze-dried *in vitro* digested samples (FDB, IFF, SFF and PRE) in a proportion of 1% (0,5 g) were dissolved in 1 mL of water and added to each fermentation bottle. In addition, negative control (C) with no sample added were also analyzed. After this, the fermentation bottles were purged with nitrogen and then incubated for 48 h at 37 °C in a shaking bath, simulating colonic lumen conditions. Aliquots of the fermented fecal samples were collected at baseline (0 h) and after 4, 8, 24 and 48 h to analyze microbial catabolites. Samples were immediately stored at -80 °C prior to analysis.

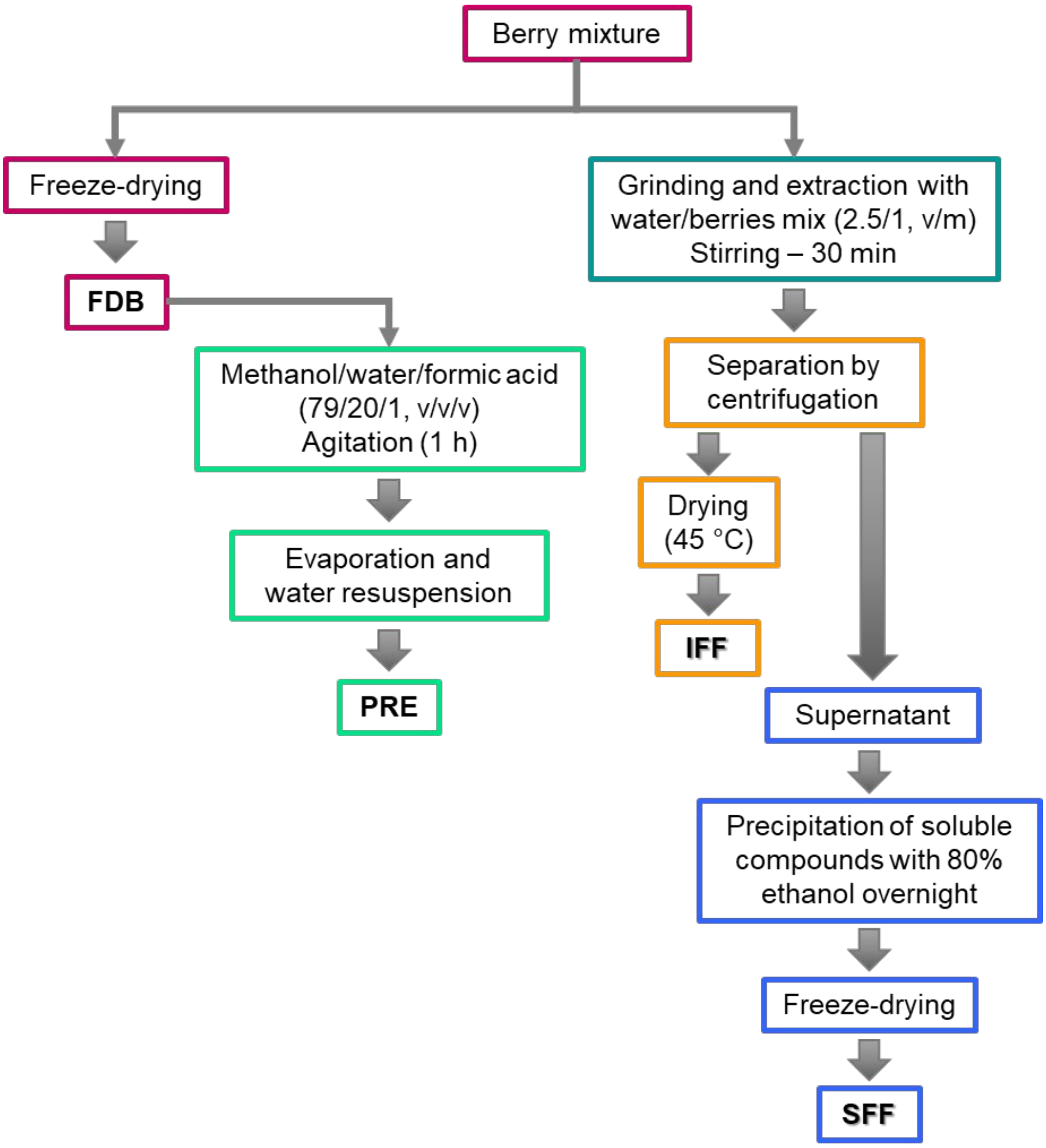

Figure S1. Flow diagram of the procedure used to obtain the different fractions: freeze-dried berries (FDB), (poly)phenol rich-extract (PRE), insoluble fiber-rich fraction (IFF) and soluble fiber-rich fraction (SFF) from the berry mixture.

Table S1. Antioxidant capacity by FRAP and ORAC (µmol TE/g of d.w.) of berry samples.

Parameters	FDB	IFF	SFF
**FRAP**	35.8 ± 0.8^{b}	53.6 ± 7.7^{a}	27.6 ± 7.8^{b}
**ORAC**	235.6 ± 3.7^{b}	249.8 ± 6.2^{a}	161.1 ± 11.0^{c}

Values are expressed as mean ± SD (n =3). Different letters (a–c) indicate significant differences (p<0.05) among the samples. Freeze-dried berries (FDB); insoluble fiber-rich fraction (IFF); soluble fiber-rich fraction (SFF); ferric reducing antioxidant power (FRAP); oxygen radical absorbance capacity (ORAC); d.w. (dry weight).

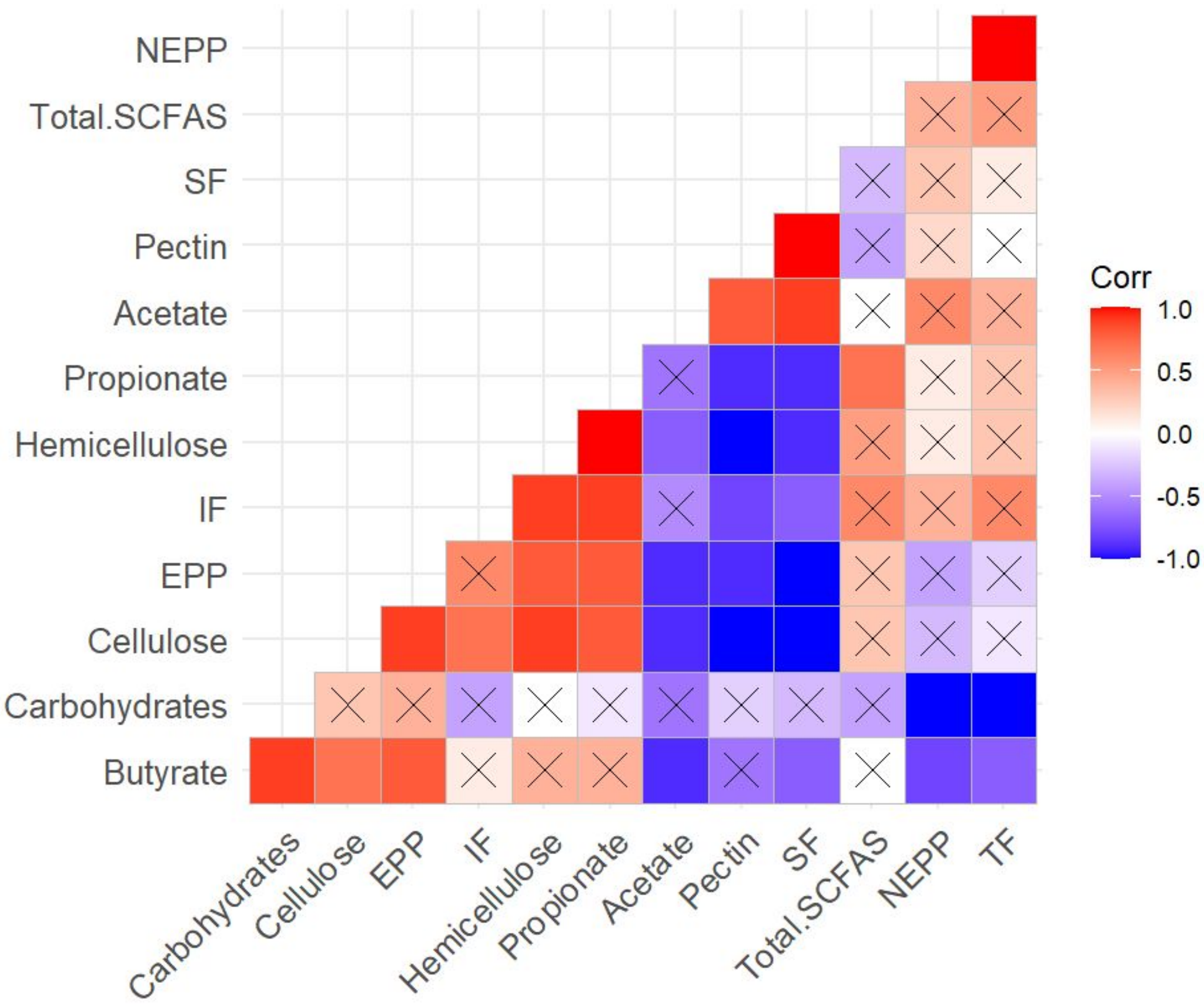

Figure S2. Correlation analysis between short chain fatty acids (SCFAs) production (acetate, propionate, butyrate and total SCFAs) and composition parameters (total fiber (TF); insoluble fiber (IF), soluble fiber (SF); cellulose; hemicellulose; pectin; carbohydrates; extractable (poly)phenols (EPP) and non-extractable (poly)phenols (NEPP)) of berry samples. "X" indicates non significant differences.

Table S2. HPLC-HR-MS based tentative identification of (poly)phenols and their microbiota-derived metabolites from *in vitro* fermentation of berry fractions in fecal slurries.

t_R (min)	Compounds	Chemical formula	Mass (m / z)		Δppm	MSIMI level*
			Theor	Expl		
2.9	3,4,5-Trihydroxybenzoic acid (Gallic acid)	C7H5O5	169.0132	169.0128	-1.578	1
4.2	3,4,5-Trihydroxybenzoic acid glucose (Monogalloyl glucose)	C13H16O10	331.0671	331.0659	-0.251	1
6.1	3,4-Dihydroxybenzoic acid	C7H6O4	153.0182	153.0184	1.273	1
7.4	3-(3',4'-Dihydroxyphenyl)acetic acid	C8H8O4	167.0338	167.0342	2.124	1
7.8	3-Hydroxy-3-(3′-hydroxyphenyl)propanoic acid	C9H10O4	181.0506	181.0496	0.413	1
8.4	4-Hydroxy-5-(3’,4’-dihydroxyphenyl)valeric acid	C11H14O5	225.0768	225.0765	3.288	2
8.6	(+)-Catechin	C15H14O6	289.0718	289.0711	1.541	1
8.8	3-(3’,4’-Dihydroxyphenyl)propanoic acid	C9H10O4	181.0495	181.0497	0.91	1
9.0	Procyanidin B2	C30H26O12	577.1351	577.1353	1.257	1
9.5	(−)-Epicatechin	C15H14O6	289.0718	289.0714	2.682	1
9.5	Ellagic acid hexoside	C20H16O13	463.0518	463.0508	0.266	2
9.6	Valoneic acid dilactone	C21H10O13	469.0049	469.0048	2.118	2
9.7	3-(3´-Hydroxyphenyl)propanoic acid	C9H10O3	165.0546	165.0544	-0.248	1
10.0	3,4,8,9,10-Pentahydroxy-urolithin (urolithin M5)	C13H8O7	275.0197	275.0192	2.076	2
10.3	5-(3’,4’-Dihydroxyphenyl)-γ-valerolactone	C11H12O4	207.0663	207.0661	4.272	1
10.4	Ellagic acid deoxyhexose	C20H16O12	447.0569	447.0561	0.711	2
10.7	3,4,8,9-Tetrahydroxy-urolithin (urolithin D)	C13H8O6	259.0248	259.0249	4.577	2
10.9	Ellagic acid	C14H6O8	300.9989	300.9990	3.443	1
11.4	3,8,9,10-Tetrahydroxy-urolithin (urolithin M6)	C13H8O6	259.0248	259.0249	4.461	2
11.7	4-hydroxy-5-(3’-hydroxyphenyl)valeric	C11H14O4	209.0819	209.0817	4.422	2
12.4	3,8,9-Trihydroxy-urolithin (urolithin C)	C13H8O5	243.0299	243.0298	4.486	2
13.5	3,8,10-Trihydroxy-urolithin (urolithin M7)	C13H8O5	243.0299	243.0298	4.362	2
13.7	3,8-Dihydroxy-urolithin (urolithin A)	C13H8O4	227.0349	227.0348	3.897	1
13.9	4-Hydroxy-5-(phenyl)valeric acid	C11H14O3	193.0870	193.0865	2.793	2

16.6	3-hydroxy-urolithin (urolithin B)	C13H8O3	211.0401	211.0390	0.092	1

*Metabolite standards initiative (MSI) metabolite identification (MI) levels1. Reference compounds were available for all compounds identified at MSI MI level 1. Summer, L. W.; Amberg, A.; Barrett, D.; Beale, M. H.; Beger, R.; Daykin, C. A.; Fan, T. W.; Fiehn, O.; Goldagre, R.; Griffin,J. L. Proposed minimum reporting standards for chemical analysis. Chemical Analysis Working Group (CAWG) Metabolomics Standards Initiative (MSI). Metabolomics 2007, 3, 211-221

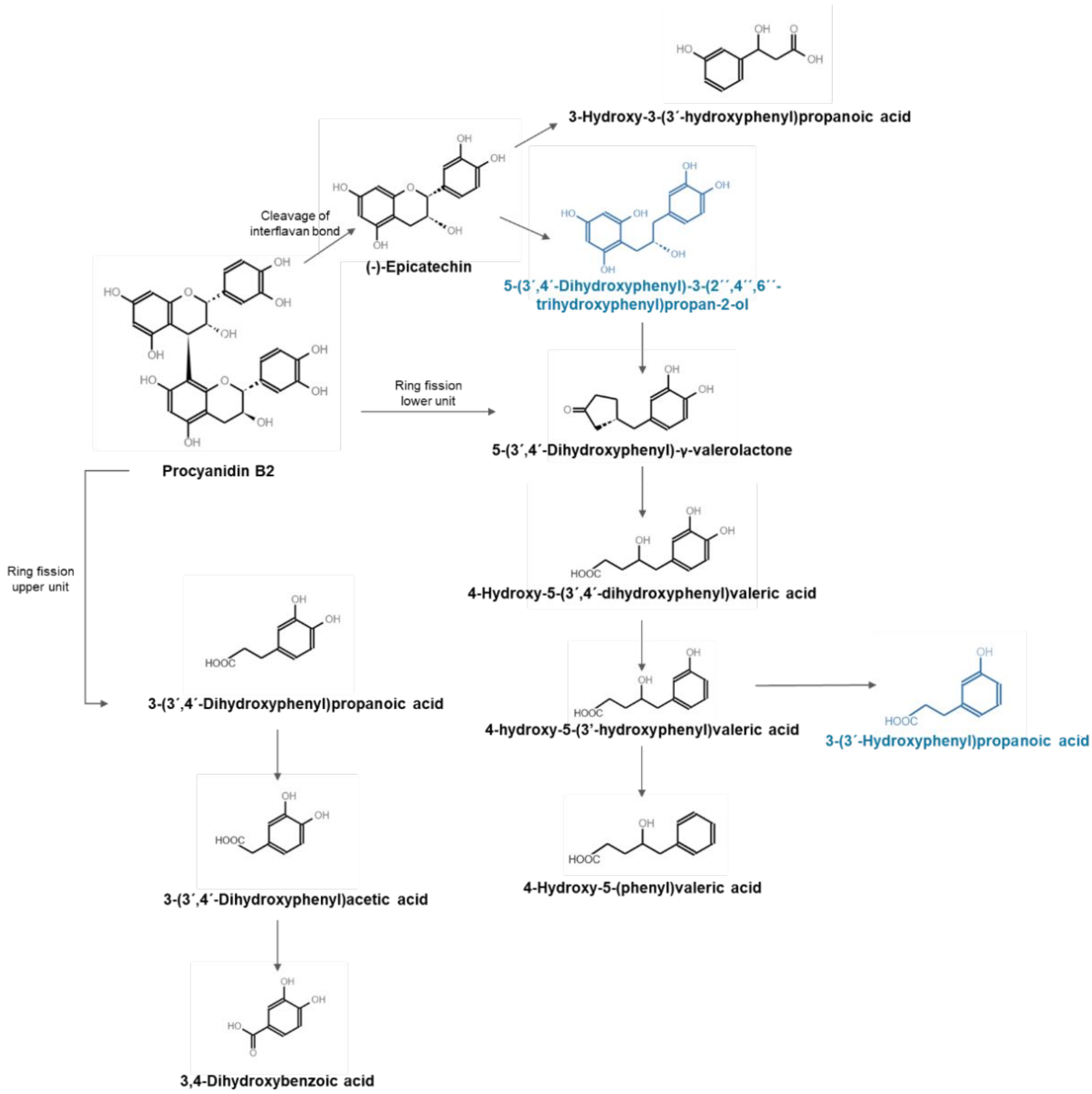

Figure S3. Proposed degradation pathway from procyanidin B2, based on Ottaviani et al. (2016), Borges et al. (2018), Sánchez-Patán et al. (2012) and Di Pede et al. (2023). Blue color indicates compounds not found in the present study.

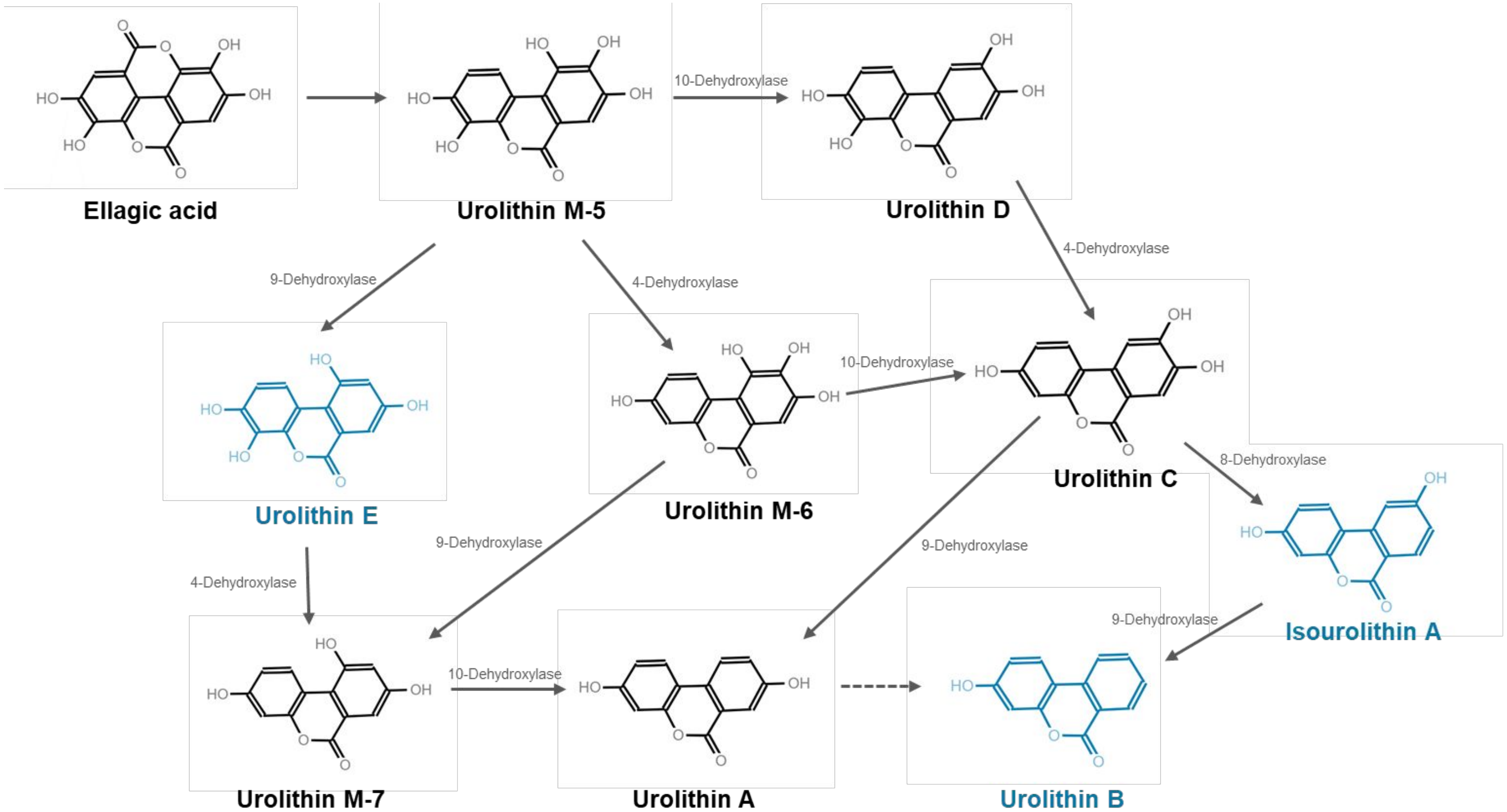

Figure S4. Proposed degradation pathway from ellagic acid, based on González-Barrio et al. (2011) and García-Villalba et al. (2019). Blue color indicates compounds not found in the present study.
